# Metagenomic characterisation of avian parvoviruses and picornaviruses from Australian wild ducks

**DOI:** 10.1038/s41598-020-69557-z

**Published:** 2020-07-30

**Authors:** Jessy Vibin, Anthony Chamings, Marcel Klaassen, Tarka Raj Bhatta, Soren Alexandersen

**Affiliations:** 1 Geelong Centre for Emerging Infectious Diseases, Geelong, VIC 3220 Australia; 2 School of Medicine, Deakin University, Geelong, VIC 3220 Australia; 3 Centre for Integrative Ecology, Deakin University, Waurn Ponds, VIC 3216 Australia; 4 Barwon Health, Geelong, VIC 3220 Australia

**Keywords:** Microbiology, Diseases

## Abstract

Ducks can shed and disseminate viruses and thus play a role in cross-species transmission. In the current study, we detected and characterised various avian parvoviruses and picornaviruses from wild Pacific black ducks, Chestnut teals, Grey teals and Wood ducks sampled at multiple time points from a single location using metagenomics. We characterised 46 different avian parvoviruses belonging to three different genera *Dependoparvovirus, Aveparvovirus* and *Chaphamaparvovirus,* and 11 different avian picornaviruses tentatively belonging to four different genera *Sicinivirus*, *Anativirus*, *Megrivirus* and *Aalivirus*. Most of these viruses were genetically different from other currently known viruses from the NCBI dataset. The study showed that the abundance and number of avian picornaviruses and parvoviruses varied considerably throughout the year, with the high number of virus reads in some of the duck samples highly suggestive of an active infection at the time of sampling. The detection and characterisation of several parvoviruses and picornaviruses from the individual duck samples also suggests co-infection, which may lead to the emergence of novel viruses through possible recombination. Therefore, as new and emerging diseases evolve, it is relevant to explore and monitor potential animal reservoirs in their natural habitat.

## Introduction

Birds and other animals can be reservoirs for zoonotic viruses that may have serious implications for human health and agriculture, for example, avian influenza A virus^1^ or severe acute respiratory syndrome coronavirus^2^. Among birds, notably wild ducks constitute a significant reservoir for viruses including, but not limited to, influenza viruses^3,4^ and coronaviruses^5,6^. Such viruses can become potential threats by spreading to other animals and humans. Thus, metagenomic characterisation of viruses from wild ducks may provide insight into viruses for which these birds may act as a natural reservoir.

Viruses are shown to co-circulate within a community leading to co-infections^6–9^ and may result in the emergence of new viruses through possible reassortment or recombination^10–13^. These novel viruses could have the potential to cause new virus disease with little prophylaxis or therapy to counteract, thus becoming a One-Health concern. However, very little is known about viruses circulating in wild ducks in Australia. Few studies have explored whether seasonal changes affect the presence and abundance of such viruses albeit efforts have been made to understand the avian virus community of Australian wild birds at a given time point^5,6^, and the spatiotemporal dynamics of highly pathogenic viruses such as influenza viruses from Australian waterfowl and shorebirds^14,15^.

Parvoviruses are amongst the smallest known DNA viruses, with virions of about 24 nm in diameter^16^. Parvoviruses are thought to have evolved to be rather host-specific, however, variants of some parvoviruses have a broader range of hosts^17^. Avian parvoviruses are associated with gastrointestinal disease, especially in juvenile birds, although they have also been isolated from healthy birds^16^. Picornaviruses are RNA viruses and one of the most diverse family of viruses, with virions of about 30 nm in diameter^18^. Like parvoviruses, most picornaviruses are thought to possess a relatively narrow host species spectrum^19^. However, some picornaviruses like the foot-and-mouth disease virus can infect a broad range of hosts such as wild and domestic ruminants and swine^20^. Some avian picornaviruses like avihepatovirus and avian encephalomyelitis virus also infect a broader host range from ducks to pigeons and other birds^21^. Avian picornaviruses can cause infections ranging from subclinical infection to clinical signs of disease such as a drop-in egg production and decreased growth^21–24^. Little is known about the diversity, host spectrum, pathogenicity, seasonal variation and factors that affect co-circulation and co-infection of avian parvo- and picornaviruses circulating in wild ducks.

The current study was designed to detect and characterise avian viruses present in Australian wild ducks by analysing faecal samples collected at various time points from a single location. Specifically, we wanted to determine the virus diversity, its abundance and seasonal variation in wild Australian ducks. As parvoviruses and picornaviruses were the most abundant and most often detected viruses^25,26^, we will here focus only on the results for these viruses.

## Results

From the initial data analysis, it became apparent that reads mapped to parvoviruses and picornaviruses were particularly abundant compared to other avian viruses in many of the samples included in the current study. Therefore, we have here only focused on the results for the parvoviruses and picornaviruses.

### Prevalence, seasonal distribution and detection of parvoviruses and picornaviruses from wild duck samples

Avian parvoviruses and picornaviruses were present in samples from each of the species of ducks captured, and all but one pooled sample contained more than one parvovirus and picornavirus. A total of 102 complete or partial parvovirus sequences (Supplementary Material 1 Parvovirus Row 1–102) and 26 complete or partial picornavirus sequences (Supplementary Material 1 Picornavirus Row 1–26) were generated from the samples analysed. When these sequences were aligned against virus reference genomes (Supplementary Material 2, Figures S1–S5), the minimum number of individual viruses these sequences possibly represented in each sample were at least 15 parvoviruses and 5 picornaviruses in the Pacific black duck samples (PBD12.16, PBD05.18 and PBD08.18), 23 parvoviruses and 3 picornaviruses from the Chestnut teal samples (CT05.18, CT08.18 and CT11.18), 2 parvoviruses and no picornaviruses from the single Grey teal sample (GT11.18) and 6 parvoviruses and 3 picornaviruses from the single Wood duck sample (WD08.18). In total, we detected and characterised sequences from at least 46 different duck parvoviruses and 11 different picornaviruses from the seven pools and three individual samples (CT08.18/11356, CT08.18/12952 and CT11.18). The minimum number of viruses and the percentage abundance of virus reads identified in these samples are given in Table 1. It should be noted that some parvoviruses and picornaviruses were excluded from the study as the number of virus reads were insignificant (< 0.0001%) or it was not possible to generate consensus sequences of at least 500 nucleotides with a reasonably good coverage (> 20) at a minimum mapping quality of 20 (Table 1).

**Table 1:** Number of avian parvoviruses and picornaviruses identified and the total abundance of viruses in each of the samples.

	Late autumn		Late winter		Late spring/early summer			
	May-18 (05.18)		Aug-18 (08.18)		Nov-18 (11.18)		Dec-16 (12.16)	
	Minimum number of viruses	Virus reads generated (%)	Minimum number of viruses	Virus reads generated (%)	Minimum number of viruses	Virus reads generated (%)	Minimum number of viruses	Virus reads generated (%)
Parvovirus								
PBD	1^?^	0.0027	**7**	**0.0522**	NA		**8**	**4.7747**
CT	1^?^	0.0002	**7**	**0.0894**	**2 (individual)**	**0.0220 (individual)**	NA	
			**14 (individual)**	**0.9400 (individual)**				
GT	NA		NA		**2**	**0.0340**	NA	
WD	NA		**6**	**0.1725**	NA		NA	
Picornavirus								
PBD	**2**	**3.1100**	**3**	**0.5100**	NA		2^?^	< 0.0001
CT	**1**	**0.0260**	**2**	**0.0002**	**0 (individual)**	**0.0000 (individual)**	NA	
GT	NA		NA		**0**	**0.0000**	NA	
WD	NA		**3**	**15.8490**	NA		NA	

The table gives the minimum number of avian parvo- and picornaviruses identified and the percentage abundance of the virus reads (%). The bold values give the data on the samples used for the current study. The underlined values with (^?^) indicate the presence of virus; however, the number of virus reads were insignificant (< 0.0001%) or no possible long consensus sequences (> 500nt with reasonable coverage of greater than 20 for a minimum mapping quality of 20) were generated. Hence these virus reads were excluded from the study. The values having (NA) indicate no sample collection on the particular time period.

The abundance and number of avian picornaviruses and parvoviruses varied considerably across the sampling time points. Parvoviruses were detected in ducks throughout the year. In winter and spring/summer samples, more different parvoviruses were detected (6–14 in late winter, 2–8 in spring/summer) compared to the autumn samples (minimum of 1 virus/pooled sample with very low abundance, i.e. 0.0002–0.0027%). However, the abundance of parvoviruses in winter and spring/summer samples (0.05–0.94% of reads in samples from late winter, 0.02–0.03% of reads in spring/summer) were low, except for the juvenile Pacific black duck December 2016 sample (4.77% of reads), which may be due to the age of the birds rather than any seasonal factor, as parvoviruses prefer multiplying host cells for their replication^27^ (Table 1). Picornaviruses, in contrast, were mainly found during late autumn to late winter months with 1–3 picornaviruses with an abundance of 0.0260–15.8490% [Table 1]. During late spring/early summer months, picornavirus reads were found in only one of the pooled samples from PBD at very low abundance (< 0.0001%) and no contigs were able to be generated from these viruses for downstream analysis.

The Pacific black duck sample from December 2016 (PBD12.16) contained the highest number of total parvovirus reads with 47,700 parvovirus reads/million reads (4.77%) and these belonged to at least 8 different parvovirus genomes (Table 1). The most highly abundant parvovirus consensus sequence was designated Pacific black duck chaphamaparvovirus 1 (PBDCPaV1/PBD12.16) and this constituted 4.27% of the generated NGS reads from this sample alone.

The Wood duck sample from August 2018 (WD08.18) contained the highest number of picornavirus reads with 158,400 picornavirus reads/million reads (15.84%) mapped to at least 3 different picornaviruses (Table 1). The most highly abundant picornavirus consensus sequence was a megrivirus designated as Wood duck megrivirus/6497nt (WDMeV/6497nt/WD08.18) which constituted 6.12% of the reads from this sample. In addition, this sample had 1,700 parvovirus reads/million reads (0.17%) which belonged to at least 6 different parvoviruses (Table 1).

### Diversity of the parvoviruses in the duck samples and their evolutionary analysis

The parvovirus sequences detected in the Australian ducks were diverse and belonged to three different genera of parvoviruses: *Dependoparvovirus, Aveparvovirus* and *Chaphamaparvovirus.* Out of the 102 assembled parvovirus consensus sequences from the duck samples, five near full-length parvovirus sequences were obtained. These included Pacific black duck adeno-associated virus (PBDAAV/PBD12.16), Pacific black duck chaphamaparvovirus 1 (PBDCPaV1/PBD12.16) and three Chestnut teal chaphamaparvoviruses (CTCPaV1/CT08.18, CTCPaV2/CT08.18/12952 and CTCPaV3/CT08.18/12952). One of the Chestnut teal chaphamaparvovirus sequences was assembled from the pooled sample (CT08.18) and two more were assembled from the individual Chestnut teal sample (CT08.18/12952).

**Figure 1:**
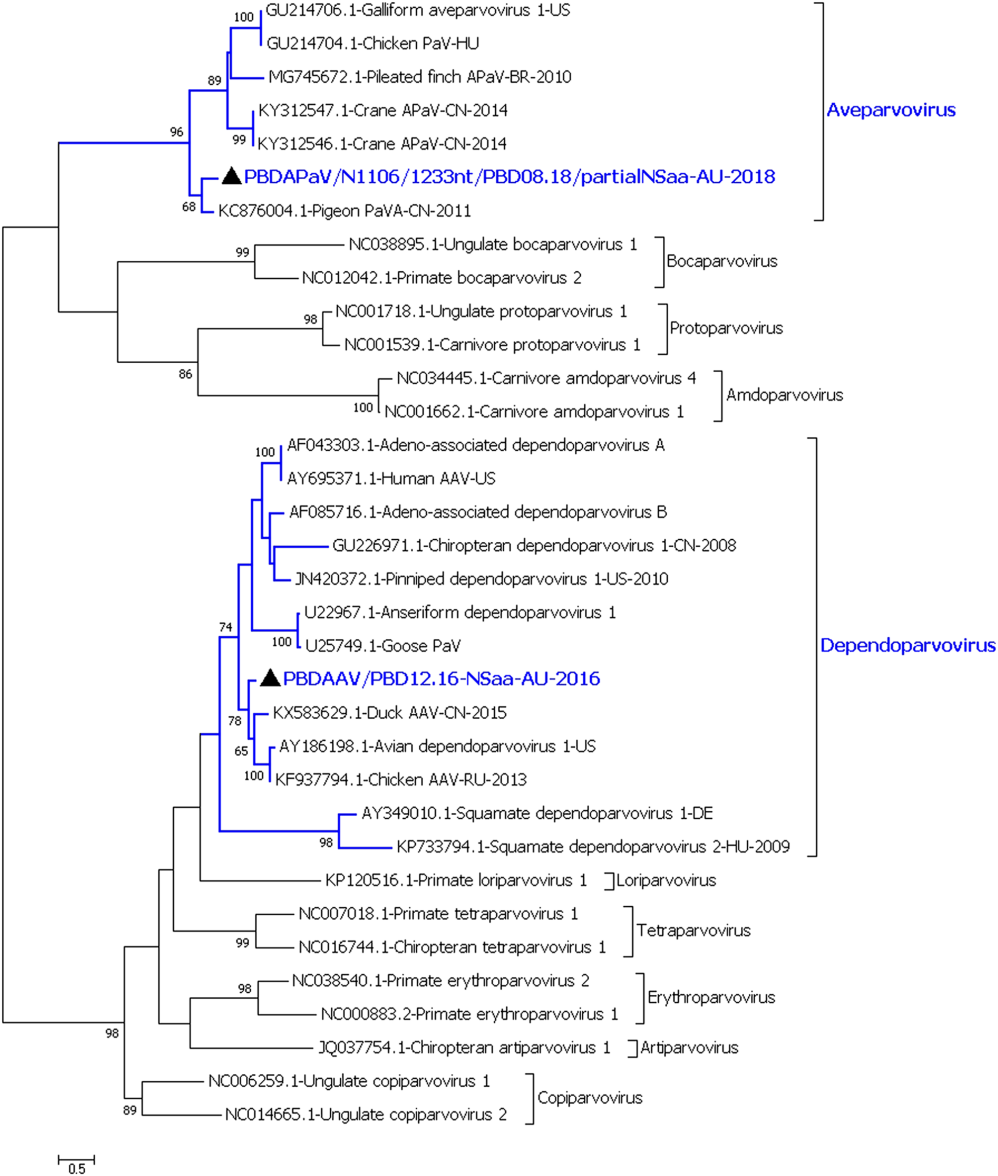
Phylogenetic analysis of partial NS1 amino acid sequences of duck parvoviruses and representative viruses from subfamily *Parvovirinae*. Parvoviruses belonging to the genera *Dependoparvirus* and *Aveparvovirus* from the subfamily *Parvovirinae* were detected in the Australian ducks from the current study*.* The evolutionary history was inferred by using the Maximum Likelihood method based on the LG+G model^66^. The analysis involved 34 amino acid sequences. All positions containing gaps and missing data were eliminated. There was a total of 235 amino acid positions in the final dataset. The robustness of different nodes was assessed by bootstrap analysis using 1,000 replicates for amino acid alignments. The numbers at the nodes represent bootstrap values and only bootstrap values at or above 60% are shown. The genera from subfamily *Parvovirinae* with viruses from the duck samples is shown in blue colour. Pacific black duck viruses are shown with black triangle.

**Figure 2:**
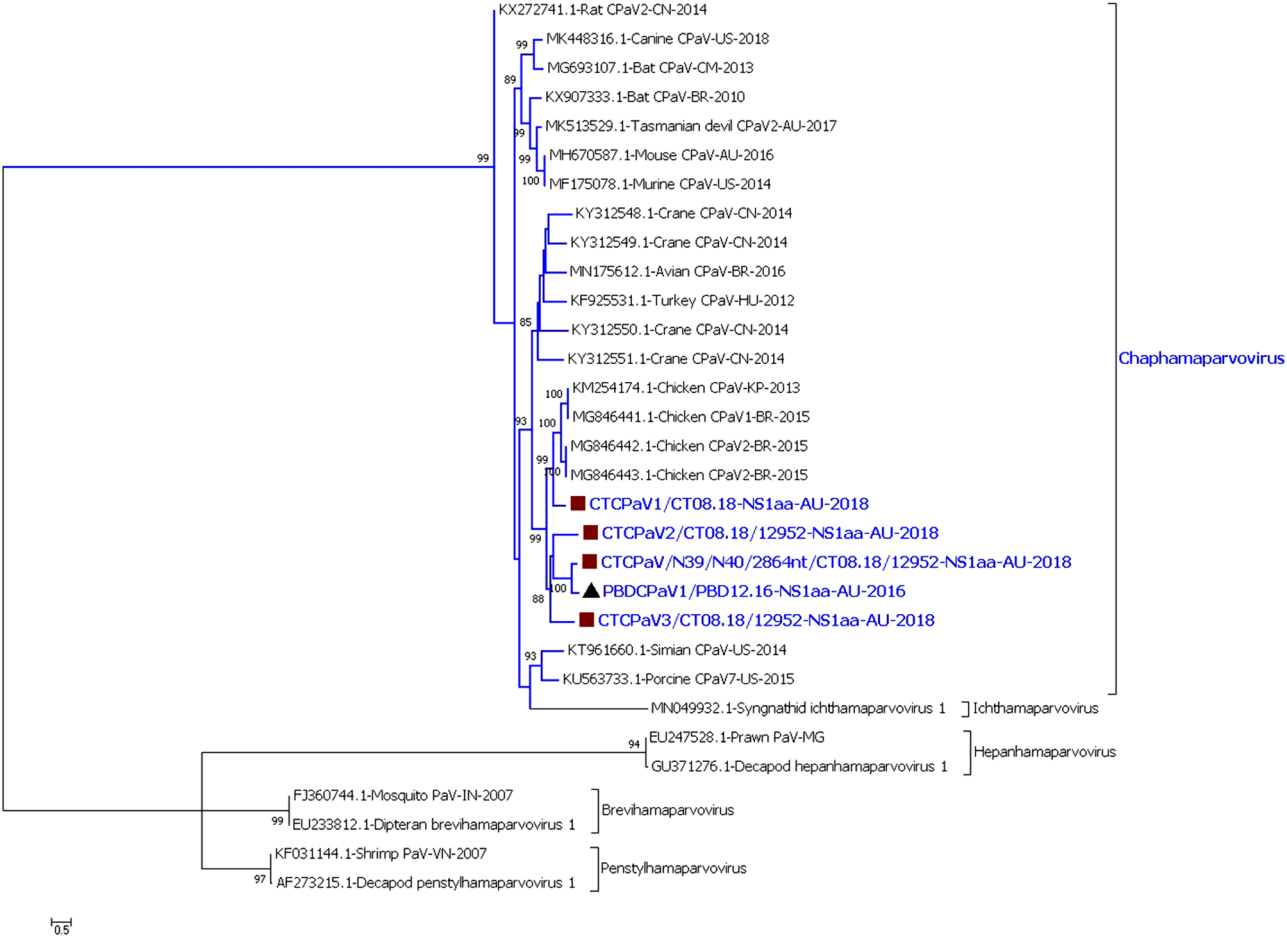
Phylogenetic analysis of non-structural amino acid sequence of duck chaphamaparvoviruses (CPaV) encoding complete NS1 protein and representative viruses from subfamily *Hamaparvovirinae*. Parvoviruses belonging to the genus *Chaphamaparvovirus* from the subfamily *Hamaparvovirinae* were detected in the Australian ducks from the current study*.* The evolutionary history was inferred by using the Maximum Likelihood method based on the LG+G+F model^66^. The analysis involved 31 amino acid sequences. All positions containing gaps and missing data were eliminated. There was a total of 442 amino acid positions in the final dataset. The robustness of different nodes was assessed by bootstrap analysis using 1,000 replicates for amino acid alignments. The numbers at the nodes represent bootstrap values and only bootstrap values at or above 60% are shown. The genus from subfamily *Hamaparvovirinae* with viruses from the duck samples is shown in blue colour. Pacific black duck viruses are shown with black triangle and Chestnut teal viruses are with brown square.

A near full-length *Dependoparvovirus* was found in the Pacific black duck pool from December 2016 [Pacific black duck adeno-associated virus (PBDAAV/PBD12.16)]. Three partial *Aveparvovirus* sequences were found in the August 2018 Pacific black duck pool. These three sequences likely came from a single virus as they each represented a different section of the aveparvovirus genome [Pacific black duck aveparvovirus (PBDAPaV/N1106/1233nt/PBD08.18, PBDAPaV/N443/650nt/PBD08.18 and PBDAPaV/N443/1497nt/PBD08.18)]. The remaining assembled parvovirus sequences from all the duck species belonged to at least 44 viruses within the genus *Chaphamaparvovirus* (CPaV) [Supplementary material 1 (Parvovirus Row 1–102)]. The generated parvovirus sequences, which included the complete non-structural protein (NS1) of the Pacific black duck adeno-associated virus and the Pacific black duck aveparvovirus encoding partial NS1 were aligned with representative parvoviruses from each genus of the subfamily *Parvovirinae* (Fig. 1). The duck chaphamaparvovirus sequences encoding the complete NS1 protein were aligned with representative parvoviruses from each genus of the subfamily *Hamaparvovirinae* (Fig. 2).

#### Phylogenetic analysis of the Pacific black duck dependoparvovirus (PBDAAV/PBD12.16)

Although a brief account of this virus was published earlier^6^, here we provide a more detailed analysis of the full-length genome of the virus as more of the genome was obtained from the latest resequencing of the sample. The PBDAAV/PBD12.16 was most similar, but distantly related to KX583629, a dependoparvovirus identified in a Muscovy duck from China in 2015^28^ with 82.5% identity shared between the NS1 proteins of these viruses (Figure S6 of the Supplementary Material 2; Table 2). The phylogenetic analysis of the NS1 protein showed that the Pacific black duck dependovirus was likely a new species within the genus *Dependoparvovirus* according to the current species definition of < 85% NS1 amino acid sequence identity established by the ICTV^29^.

**Table 2:** The amino acid comparison of the avian parvoviruses and picornaviruses from the Australian duck samples.

Virus | NCBI Accession number	Protein	Percentage similarity to its nearest relative
**Parvovirus**		
PBDAAV/PBD12.16 | MT247729	NS1	(KX583629) Muscovy duck AAV^28^: 82.5%
PBDAPaV/N1106/1233nt/PBD08.18 | MT247755	Partial NS1	(KC876004) Pigeon APaV^33^: 67.7%
PBDCPaV1/PBD12.16 | MT247730	NS1	(MT247765) CTCPaV/N39/N40/2864nt/CT08.18/12952: 82.2%
CTCPaV1/CT08.18 | MT247758	NS1	(MG846442 and MG846443) Chicken CPaV: 69.6%
CTCPaV2/CT08.18/12952 | MT247759	NS1	(MT247758) CTCPaV1/CT08.18: 57.1%
CTCPaV3/CT08.18 /12952 | MT247760	NS1	(MT247758) CTCPaV1/CT08.18: 60.4%
CTCPaV/N39/N40/2864nt/CT08.18/12952 | MT247765	NS1	(MT247730) PBDCPaV1/PBD12.16: 81.9%
**Picornaviruses**		
PBDAaV/7615nt/PBD08.18 | MT247844	RdRp, Peptidase C3, Helicase and capsid	(KJ000696) Duck Aalivirus A: 82.8%
PBDAaV/4182nt/PBD05.18 | MT247842	RdRp, Peptidase C3 and Helicase	(KJ000696) Duck Aalivirus A: 98.8%
CTAaV/N55/2515nt/CT05.18 | MT247835	RdRp, Peptidase C3 and Helicase	(KJ000696) Duck Aalivirus A: 65.5%
PBDSV-like virus/4792nt/PBD05.18 | MT247840	RdRp, Peptidase C3 and Helicase	(KF979331) Chicken picornavirus 1: 61.4%
PBDAnV-like virus/5691nt/PBD08.18 | MT247843	RdRp, Peptidase C3, Helicase, 2B and capsid	(AY563023) Duck Anativirus A: 52.8%
PBDMeV/2821nt/PBD08.18 | MT247849	RdRp and Peptidase C3	(MK204391) Duck megrivirus: 99.2%
WDMeV/N9/2933nt/WD08.18 | MT247851	RdRp and Peptidase C3	(MT247854) WDMeV/4874nt/WD08.18: 99.5%
WDMeV/N87/2221nt/WD08.18 | MT247852	RdRp and Peptidase C3	(MT247854) WDMeV/4874nt/WD08.18: 96.8%
WDMeV/N12/4874nt/WD08.18 | MT247854	RdRp, Peptidase C3 and Helicase	(MT247851) WDMeV/2933nt/WD08.18: 99.7%

The table gives the percentage similarity of the NS1 or the RdRp encoded protein of the duck parvoviruses and picornaviruses consensus sequences respectively. 1st column provides the virus sequences generated from the duck sample, 2nd column gives the analysed protein encoded by the sequence, 3rd column provides the data on the identity of the protein to its nearest relative. All the data given in this table is calculated from the distance data table generated after the phylogenetic analysis.

The phylogenetic analysis of the capsid protein encoded by the duck parvoviruses and picornaviruses was carried out and the genetic distance calculated to find the closest relative sequence (Column J of Supplementary material 1). The phylogenetic analysis of the virus capsid protein agreed with their NS1 phylogenetic analysis (data not shown). The capsid protein of the PBDAAV/PBD12.16 virus like the NS1, was most similar, but distantly related to KX583629 Muscovy duck dependoparvovirus but with 70.9% similarity. The greatest amount of genetic variability was in the VP3 region of the capsid protein from ~ 450^th^ to 600^th^ amino acid, which has been recognised as the most variable region of the dependoparvovirus capsid sequence^30,31^. The PBDAAV/PBD12.16 also contained an open reading frame of 489 nucleotides (2792nd to 3280th nucleotide) that likely encoded an assembly activation protein required for capsid packaging^32^. Although we cannot definitively say whether this dependoparvovirus was able to replicate/infect with or without a helper virus, it phylogenetically grouped with other helper-dependent adeno-associated viruses and this sample contained an avian adenovirus^6^.

#### Phylogenetic analysis of the Pacific black duck aveparvovirus (PBDAPaV/PBD08.18)

The three partial sequences from the Pacific black duck aveparvovirus from the August 2018 sample (PBDAPaV/PBD08.18) encoded a partial non-structural protein 1 (NS1), a partial hypothetical protein (HP) and a partial capsid protein, all most likely from a single virus. The phylogenetic analysis of the partial non-structural amino acid sequences showed that it was distantly related to KC876004.1, a pigeon Parvovirus A from China in 2011^33^ with 67.7% similarity over 387 amino acids and less similar to other known aveparvoviruses (Figure S7 of the Supplementary material 2 and Table 2). The NS1 and the capsid protein analysis shows that the PBDAPaV/PBD08.18 virus could be classified as a new species within the genus *Aveparvovirus*^29^.

#### Phylogenetic analysis of the chaphamaparvoviruses

**Figure 3:**
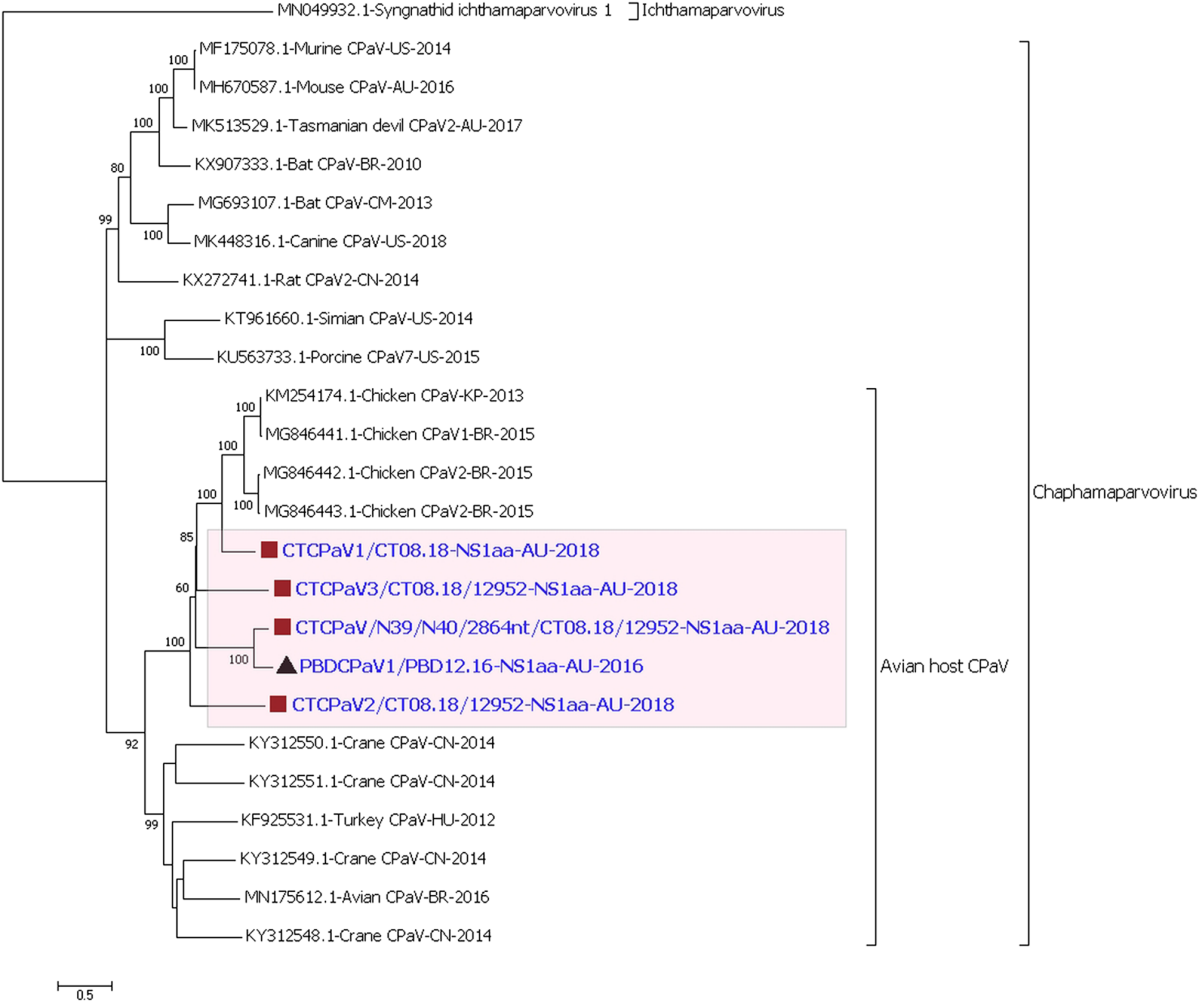
Phylogenetic analysis of the non-structural amino acid sequence of duck chaphamaparvoviruses (CPaV) encoding complete NS1 protein. The evolutionary history was inferred by using the Maximum Likelihood method based on the model LG+G+I+F^66^. The analysis involved 25 amino acid sequences. All positions containing gaps and missing data were eliminated. There were a total of 525 positions in the final dataset. The robustness of different nodes was assessed by bootstrap analysis using 1,000 replicates for amino acid alignments. The numbers at the nodes represent bootstrap values and only bootstrap values at or above 60% are shown. The highlighted section shows duck CPaV lineages/clusters. Pacific black duck virus is shown with (black triangle) and Chestnut teal viruses are shown with (brown square).

The four near full-length chaphamaparvovirus sequences and one partial sequence which contained the full NS1 were more closely related to each other (57.1–82.2% similarity) and more distantly related to currently known viruses in the NCBI dataset (56.3–69.6% similarity). They, however, still belonged to an avian lineage within the chaphamaparvovirus genus (Table 2; Fig. 3). For example, the 688 amino acid sequence of the NS1 protein of the PBDCPaV1/PBD12.16 had only 82.2% similarity to CTCPaV/N39/N40/2864nt/CT08.18/12952, and was even less similar (45.0–58.1%) to other chaphamaparvovirus NS1 amino acid sequences. Both the phylogenetic and BLAST analyses of the NS1 (Figure S8-S10 of the Supplementary Material 2) and capsid protein (Column J of the Supplementary Material 1) amino acid sequence showed that our duck chaphamaparvoviruses were distantly related to any other avian host-associated chaphamaparvovirus previously described, and form an evolutionary duck clusters/lineages closest to the chicken chaphamaparvoviruses. Each of the duck sub-clusters included viruses from more than one host species indicating that these parvovirus lineages may either have evolved into individual strains or species that may have the ability to infect closely related duck species. The detection and characterisation of multiple chaphamaparvoviruses strains or species from individual samples also suggested co-infection, which is discussed below.

### Diversity of the picornaviruses in the duck samples and their evolutionary analysis

**Figure 4:**
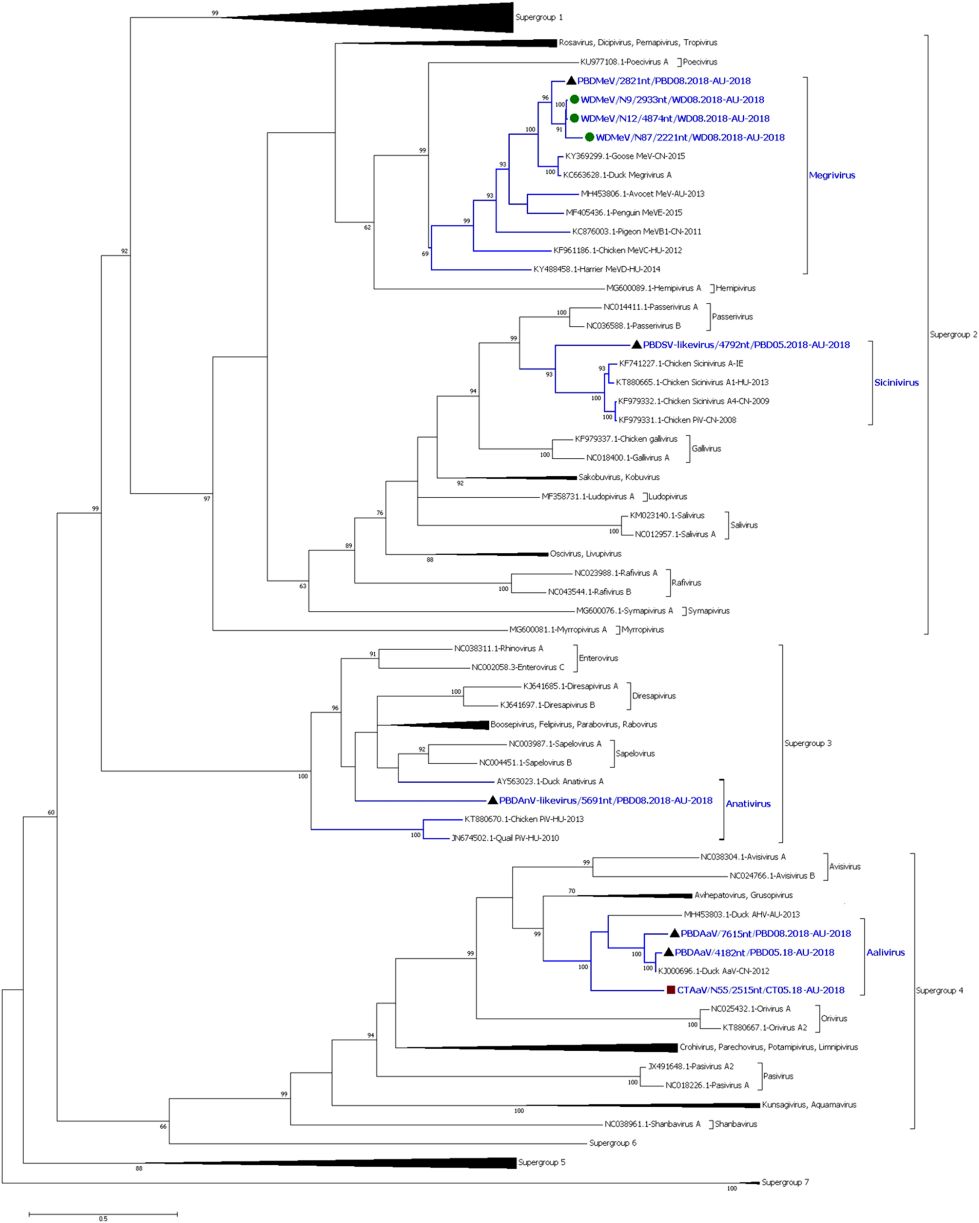
Phylogenetic analysis of the partial RdRp region of the duck picornaviruses and of representative viruses from *Picornaviridae* family. The picornavirus sequences detected in the Australian ducks from the current study tentatively belong to four different genera: *Sicinivirus*, *Anativirus*, *Megrivirus* and *Aalivirus.* The evolutionary history was inferred by using the Maximum Likelihood method based on the LG+G+I^66^ model. The analysis involved 129 amino acid sequences. All positions containing gaps and missing data were eliminated. There were a total of 313 amino acid positions in the final dataset. The robustness of different nodes was assessed by bootstrap analysis using 1,000 replicates for amino acid alignments. The numbers at the nodes represent bootstrap values and only bootstrap values at or above 60% are shown. Each of the genus from *Picornaviridae* is shown with viruses from the duck samples in blue colour. Pacific black duck viruses are shown in black triangle, Chestnut teal viruses in brown square and Wood duck viruses in green circle. Supergroup 1 consists of viruses from genus *Ailurivirus, Aphthovirus, Bopivirus, Cardiovirus, Cosavirus, Erbovirus, Hunnivirus, Malagasivirus, Mischivirus, Mosavirus, Mupivirus, Senecavirus, Teschovirus, Torchivirus* and *Tottorivirus*. Supergroup 5 consists of viruses from genus *Crahelivirus, Fipivirus, Gruhelivirus, Hepatovirus, Rohelivirus* and *Tremovirus*. Supergroup 6 contains virus from the genus *Harkavirus*. Supergroup 7 consists of virus from the genus *Ampivirus.*

Phylogenetic analysis and maximum likelihood trees of the amino acid sequences of the partial polyprotein (PP) from the 26 picornavirus consensus sequences identified viruses tentatively belonging to the genera *Sicinivirus*, *Anativirus*, *Megrivirus* and *Aalivirus* (Fig. 4)*.* The tentative avian siciniviruses included two sequences likely belonging to a single Pacific black duck sicinivirus-like virus from the August pool (PBDSV-like virus/PBD05.18 of 4111nt and 4768nt long) and 1 short sequence from a Chestnut teal sicinivirus-like virus from the August pool (CTSV-like virus/505nt/CT08.18). One sequence from a Pacific black duck anativirus-like virus from August pool (PBDAnV-like virus/5691nt/PBD08.18) was identified and was the only anativirus-like virus found in the duck samples. The avian megriviruses from the duck samples included five sequences likely belonging to a single Pacific black duck megrivirus found in the August pool (PBDMeV/PBD08.18 of 3747nt, 455nt, 876nt, 1572nt and 2821nt long), seven sequences likely from three Wood duck megriviruses found in the August pool (WDMeV/WD08.18 of 6497nt, 2933nt, 2221nt, 1296nt, 4874nt, 622nt and 1071nt long) and two sequences likely from 1 Chestnut teal megrivirus from August pool (CTMeV/CT08.18 of 583nt and 363nt long). The avian aaliviruses from the duck samples included 2 Pacific black duck aaliviruses (PBDAaV/PBD05.18 of 4768nt and 4182nt long and PBDAaV/7615nt/PBD08.18) and 1 Chestnut teal aalivirus (CTAaV/CT05.18 of 242nt, 1084nt, 654nt, 2010nt and 2515nt long).

The generated partial picornavirus sequences were analysed using ORFfinder, BLAST and MEGA to determine the general position and homology of the sequences and is shown in Figure S5 in the Supplementary Material 2. The sequence WDMeV/6497nt/WD08.18 overlapped two other partial genome sequences WDMeV/N9/2933nt/WD08.18 and WDMeV/N87/2221nt/WD08.18. However, because of the short overlapping sequence (123 nucleotides for WDMeV/N9/2933nt/WD08.18 and 32 nucleotides for WDMeV/N87/2221nt/WD08.18) an appropriate conclusion on which partial sequence forms the 3′ end of this particular megrivirus could not be established. The percentage similarity between the WDMeV/N9/2933nt/WD08.18 and WDMeV/N87/2221nt/WD08.18 sequences to each other (90.0% in nucleotide level and 96.6% in amino acid level) did suggest that these two sequences were derived from two individual megriviruses, possibly sharing the same structural protein sequence but different non-structural proteins. This hypothesis was supported by the combined virus read abundances of the two non-structural sequences of the WDMeV (3.48% for the WDMeV/N9/2933nt/WD08.18 and 2.5% for the WDMeV/N87/2221nt/WD08.18) adding up to the virus read abundance of the structural sequence of WDMeV/6497nt/WD08.18 (6.12%).

#### Phylogenetic analysis of Pacific black duck anativirus-like virus (PBDAnV-like virus/PBD08.18)

The phylogenetic analysis of the PBDAnV-like virus/PBD08.18 was carried out on the amino acid sequence of the polyprotein encoded by the generated partial genome consensus sequence PBDAnV-like virus/5691nt/PBD08.18. The 1896 amino acids of the PBDAnV-like virus consensus sequence that encodes the capsid, 2B protein, RNA helicase and the RdRP were only 52.8% similar to AY563023.1; a duck anativirus A from Taiwan^22^ and even less similar (51.2–51.6%) to other anativiruses (Figure S11 of the Supplementary material 2).

#### Phylogenetic analysis of sicinivirus-like viruses

The phylogenetic analysis of the Pacific black duck sicinivirus-like virus (PBDSV-like virus/PBD05.18) was carried out on the amino acid sequence of the polyprotein encoded by the two partial genome consensus sequences. The 1,495 amino acids of the PBDSV-like virus/4792nt/PBD05.18 consensus sequence that encodes the RNA helicase, Peptidase C3 and RdRp of the sicinivirus was only 61.4% similar to KF979331.1, a chicken picornavirus 1 from Hong Kong in 2008^34^ and even less similar to other siciniviruses (Figure S12 of the Supplementary material 2). The PBDSV-like virus/4111nt/PBD05.18 consensus sequence that encoded the capsid region of the sicinivirus was only 63.0% similar over 1,020 amino acids to MG846480.1, a partial chicken sicinivirus sequence from Brazil in 2015.

The Chestnut teal sicinivirus-like virus (CTSV-like virus/505nt/CT08.18) was found to be different from the PBDSV-like virus/PBD05.18 with 88.3% similarity at the nucleotide level (BLASTN) and 97.6% similarity at the amino acid level (BLASTP).

#### Phylogenetic analysis of aalivirus

**Figure 5:**
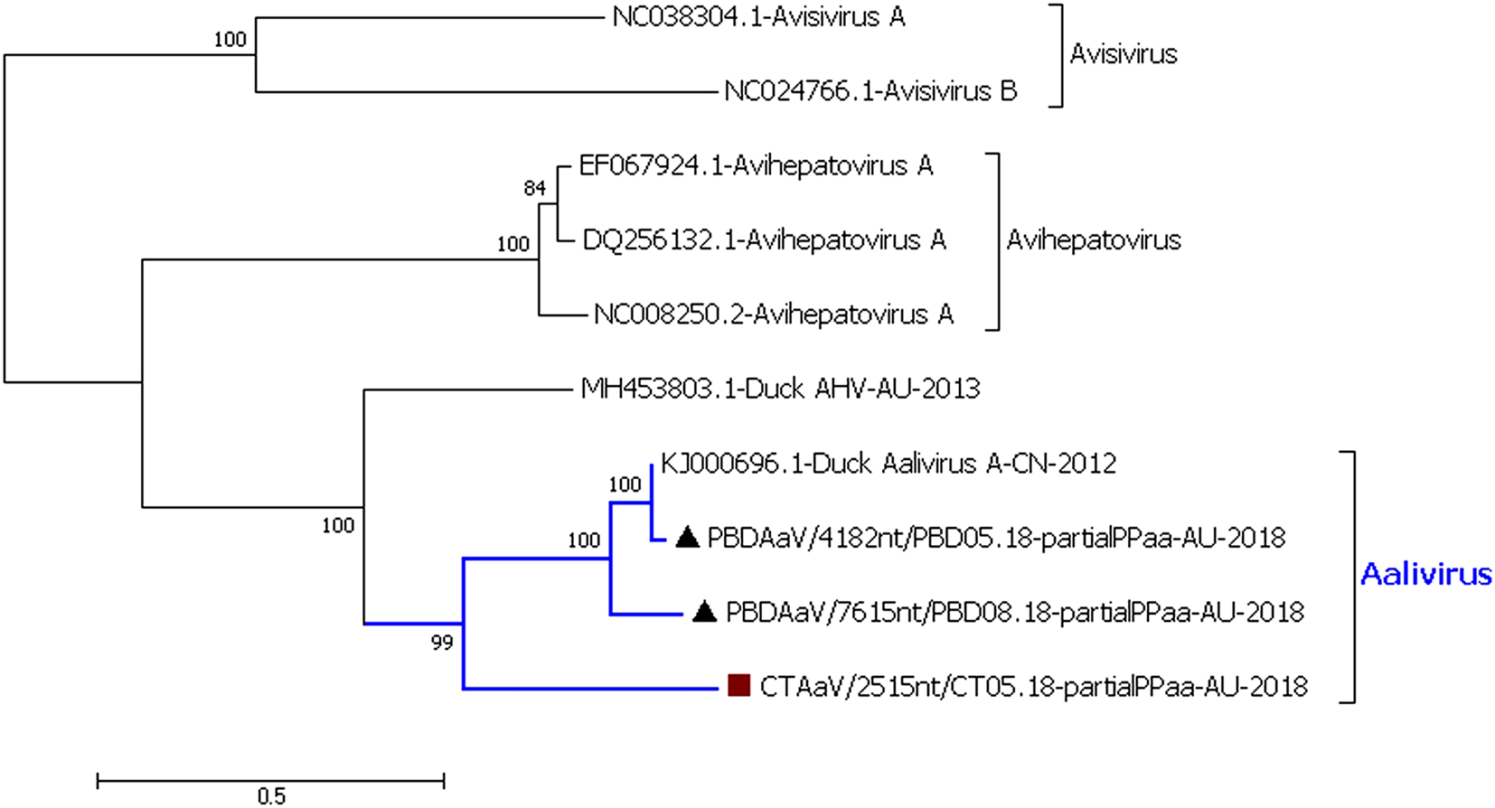
Phylogenetic analysis of the amino acid sequence of the RdRp, Helicase, Peptidase C3 of Aalivirus (AaV) sequences. Phylogenetic analysis shows three new species in the genus *Aalivirus.* The evolutionary history was inferred by using the Maximum Likelihood method based on the model LG+G^66^. The analysis involved 10 amino acid sequences. All positions containing gaps and missing data were eliminated. There were a total of 794 amino acid positions in the final dataset. MH453803.1 Duck Avehepatovirus-like virus is an unassigned virus. The robustness of different nodes was assessed by bootstrap analysis using 1,000 replicates for amino acid alignments. The numbers at the nodes represent bootstrap values and only bootstrap values at or above 60% are shown. Pacific black duck viruses are shown in black triangle and Chestnut teal viruses in brown square.

The polyprotein of the longest aalivirus sequence from the Pacific black duck May 2018 pool (PBDAaV/7615nt/PBD08.18) was distantly related to the only member of the genus *Aalivirus*, Aalivirus A (GenBank Accession: KJ000696.1)^35^, from a domestic Pekin duck from China in 2012, with 82.8% similarity. It was even less similar (51.1–62.4%) to other picornaviruses, including MH453803, an Avihepatovirus-like virus from a dabbling duck sample from Victoria in 2013^36^ (Figure S13 of the Supplementary material 2). The amino acid sequence alignment of the RNA helicase, Peptidase C3 and RdRp of three aalivirus sequences generated from the Pacific black duck and Chestnut teal samples, (PBDAaV/4182nt/PBD05.18 and CTAaV/N55/2515nt/CT05.18 along with PBDAaV/7615nt/PBD08.18) showed that the PBDAaV/4182nt/PBD05.18 consensus sequence was more closely related to KJ000696.1 Aalivirus A with 98.8% similarity, while CTAaV/N55/2515nt/CT05.18 was more distantly related to both the Pacific black duck aaliviruses and KJ000696.1 Aalivirus A (65.5%) (Fig. 5). The structural protein coding sequence of the PBDAaV/4768nt/PBD05.18 was 92.4% similar to KJ000696.1 Aalivirus A, compared to its non-structural counterpart PBDAaV/4182nt/PBD05.18. The amino acid variations in the P1 region of the PBDAaV from both PBD05.18 and PBD08.18 samples suggest that these viruses could potentially have adapted to infect wild Pacific black ducks/mallards rather than the closely related domestic Pekin duck. Both the polyprotein amino acid sequence phylogenetic analysis and individual BLAST of the sequences showed that our duck aaliviruses belong to the newly formed genus *Aalivirus* that at present has only one virus^18^.

#### Phylogenetic analysis of megrivirus:

The phylogenetic analysis of the Peptidase C3 and RdRp region of the megriviruses showed that our duck megriviruses form a cluster/lineages related to other duck and geese megriviruses (Figure S14-S15 of the Supplementary material 2). The Pacific black duck megrivirus was closely related to MK204391.1 Pacific black duck megrivirus from Australia in the year 2012^37^ with 99.2% similarity over 799 amino acids in RdRp region and with 98.7% similarity over 1,051 amino acids in capsid region. The Wood duck megriviruses were 96.8–99.5% similar to each other in the RdRp region than to other megriviruses from the NCBI dataset. As stated above, two RdRp partial sequences that overlap one long capsid encoding partial WDMeV genome sequence were characterised. The phylogenetic analysis of the partial capsid protein coding region of the Wood duck megriviruses may indicate an evolutionary recombination event as discussed below (Figure S15 in the Supplementary material 2).

### Upstream protein-coding region (uORF) of picornaviruses and chaphamaparvoviruses

Recently, the presence of an upstream protein-coding region (uORF) has been described and the expressed protein purified for some viruses belonging to the virus family *Picornaviridae*^38^. Interestingly, an uORF (as ORF1) has also recently been shown in silico to be present in some chaphamaparvoviruses^39^. Consequently, we looked for the presence of an uORF in our duck picornavirus and chaphamaparvovirus sequences using the NCBI ORFfinder (Column D of Supplementary material 1). These uORF present in some of our sequences could potentially encode a protein of 65–156 amino acids which overlaps the much larger NS1 ORF of the chaphamaparvoviruses or polyprotein of the picornaviruses. Here, for some of the parvovirus sequences (for example, CTCPaV1/CT08.18), a TATA box (promoter element) was found to be upstream of the uORF region, which indicate that this protein could potentially be expressed (Column K of Supplementary material 1).

### Additional virus sequence features

All the generated consensus sequences were also bioinformatically analysed further for the detection of possible motifs and nuclear translocation signals. Various motifs such as helicase (for example, at 253–438 aa of NS1 of PBDCPaV1/PBD12.16), bipartite nuclear localisation signal (such as RRNYGADRAGSARTRGR in the NS1 of PBDCPaV1/PBD12.16), Arginine rich region, Glycine-rich region, Serine-rich region, Ribosomal protein S14 signature, Neutral zinc metallopeptidases, Zinc finger C2H2 type domain, C-type lectin domain, AAA+ superfamily and Malic enzyme NAD binding domain were detected in some of the parvovirus and picornavirus amino acid sequences (Column K of Supplementary Material 1). The presence of these features at the expected location in the generated consensus sequences is an additional confirmation on the characterised viruses.

### Co-circulation and co-infection of viruses

The current study detected and partially characterised several parvoviruses and picornaviruses from the wild duck samples. As explained above, more than one parvovirus was found to be present in any one duck sample (for example, presence of at least 12 parvoviruses in CT08.18/12952 individual sample, 2 parvoviruses in CT08.18/11356 individual sample and at least 7 parvoviruses in the Chestnut teal August 2018 pooled sample) suggesting not only simultaneous co-circulation but indeed also co-infection. Like for the parvoviruses, the results for picornaviruses detected in the current study also suggested co-circulation and most likely co-infection. For example, in May 2018, aaliviruses were found in both the Pacific black duck and Chestnut teal samples while in August 2018, megriviruses were found in the Pacific black duck, Wood duck and Chestnut teal sample. This could suggest a seasonal co-circulation variation in the avian virome.

## Discussion

The ecological and biological characteristics of birds enable them to play a major role in the emergence of new viruses and cross-species transmission^40^. Ducks are described as “trojan horses” for viruses such as influenza viruses^3^. They can disseminate pathogenic viruses, showing few or no signs of disease. Ducks are found in nearly all aquatic habitats and can shed and spread viruses through both the respiratory and intestinal tracts^3^. These facts accentuate the importance of the current study in conducting surveillance for potential virus threats to wildlife and humans from wild ducks.

The current study shows that common wild Australian ducks (PBD, CT, GT and WD) carry a diverse number of avian parvoviruses and picornaviruses. We detected and partially sequenced around 46 parvoviruses and 11 picornaviruses from the sampled ducks, with the majority of the viruses being genetically different (up to approximately 70% in amino acid sequences of partial polyprotein of picornaviruses or NS1 of parvoviruses) from the other currently known viruses in the NCBI dataset. Some of the viruses described here were found in high abundance in the sample suggesting active infection at the time of sampling.

The duck parvoviruses and picornaviruses found varied not only in virus composition across species and time but also in their abundances, despite the ducks sharing the same habitat at the times of sampling. Duck parvoviruses were detected throughout the year, which could be because of the high virus stability exhibited by parvoviruses^41^. Nevertheless, 10–100 times more parvovirus reads were found during winter and spring/summer samples compared to the autumn samples. Human parvoviruses like Parvovirus B19 that causes fifth disease or “slapped cheek” was shown to be more prevalent during these seasons also^42^. However, in the case of animal parvoviruses, very few studies have been conducted to determine their seasonal prevalence, especially from Australia^43,44^. The abundance of parvovirus was high in the juvenile Pacific black duck samples compared to their adult counterparts, which suggests that these duck chaphamaparvoviruses prefer juvenile hosts like any other parvovirus^27^. Duck picornaviruses were mainly found during late autumn to late winter months. To the best of our knowledge, the current study is the first to analyse the seasonal abundance of both duck parvoviruses and picornaviruses, especially from Australia.

The duck parvoviruses and the picornaviruses identified and characterised in this study showed relative similarity to other avian viruses from their respective genera. The dependoparvovirus, anativirus-like virus, aalivirus and the megrivirus characterised from the Australian duck samples had the closest similarity to other duck viruses. The aveparvovirus, chaphamaparvovirus and sicinivirus-like virus from the Australian duck samples had the closest similarity to other avian viruses from chickens or pigeons. The duck chaphamaparvoviruses from the current study consistently formed duck clusters/lineages separate to chaphamaparvoviruses from other avian hosts. Each of the sub-clusters included viruses from more than one duck species indicating that these parvovirus lineages may have evolved into individual strains or species that may have the ability to infect closely related duck species. It is also to be noted that the duck aveparvovirus and the duck sicinivirus-like virus from the current study are the first duck viruses identified in their respective genus. As per the International Committee on Taxonomy of Viruses (ICTV) for all the genera under the subfamily *Parvovirinae* generally, the species demarcation criterium is to have the identity of NS1 protein amino acid sequences < 85%^16^. The picornaviruses genus demarcation criteria are to exhibit significant divergence on orthologous proteins < 66% of the capsid and < 64% of the helicase, peptidase C3 and RdRp^18^. The duck parvoviruses, the Pacific black duck sicinivirus-like virus and the Pacific black duck anativirus-like virus described here, fulfil these criteria and can be considered as new or novel virus species based on the genome sequence analysis. The detection of more than 30 new viruses, that are not previously described, belonging to two virus families from four duck species collected at three-time points indicates inadequate surveillance studies on wild ducks.

Evidence of virus co-circulation has been found from the faecal samples collected from different duck species at the same time point and location. For example, the presence of megriviruses and chaphamaparvoviruses in the faecal samples of Pacific black ducks, Wood ducks and Chestnut teals collected in August 2018 from the same location indicates virus co-circulation. In addition, the presence of aalivirus in both Pacific black ducks and Chestnut teals in May 2018 also indicates its co-circulation. However, these viruses were different in nucleotide and amino acid level even within the species level.

The simultaneous detection of related viruses in the individual sample indicates co-infection, which may lead to recombination. For example, the presence of two different non-structural Wood duck megrivirus sequences with only identical structural sequence (WDMeV/6497nt/WD08.18) leads to the speculation of two Wood duck megriviruses having the same structural protein but with different non-structural protein coding regions. This could be because of possible virus recombination^45,46^ occurring in avian host species during co-infections or due to the occurrence of gradual mutation during replication. Another example is that of the Pacific black duck aalivirus (PBDAaV/PBD05.18) showing 98.0% similarity to the KJ000696.1 Aalivirus A from China in its RdRp region while having only 92.3% similarity in its structural coding region. This suggests that this virus may have adapted to infect wild Pacific black ducks/mallards through possible recombination or gradual mutation. However, further studies are required to understand the evolution of these viruses and how that may enable cross-species infection.

The NGS data with a high number of virus reads for some of the duck samples imply the presence of an active infection and suggest a potential health impact on the birds. It should be noted that at the collection time point no close physical examination, such as the presence of any histopathological lesions, was conducted to detect any clinical signs of disease. The high viral load detected in some of the bird samples, with the caveat that all faecal samples were subjected to virus enrichment and nucleic acid amplification, suggests substantial use of infected host cell machinery by the virus for its multiplication and expression, and consequently the presence of an active infection and potential adverse effects on cells and host. For example, with respect to chaphamaparvoviruses, the identification of mouse kidney parvovirus from mice with inclusion body nephropathy^47^, the identification of cachavirus from dogs with diarrahea^48^ and the isolation of various chaphamaparvoviruses from different host faecal samples^23,49^ suggesting possible infection in the gastrointestinal area, does imply that these viruses could be pathogenic and may cause diseases in their host species. The high abundance of chaphamaparvoviruses in the juvenile Pacific black duck sample (PBD12.16) compared to their adult counterpart (PBD05.18 and PBD08.18) could also suggest that these duck chaphamaparvoviruses prefer juvenile hosts for their active infection and multiplication^27^.

Another example is the 15.8% viral abundance of megriviruses in the Wood duck WD08.18 sample. Megrivirus can cause hepatitis and enteritis in turkeys^21^. These symptoms in ducks, if caused by the virus, cannot be determined without close pathological examination. It has been previously shown that during autumn–winter seasons, due to limited pasture availability and low temperature, there is a possibility of body fat loss in birds^50^, which in turn may lead to reduced immunity^50,51^. This could be a factor that may account for susceptibility to virus infections. The abundance of the virus particles in these samples without the indication of any visible clinical signs of disease, as noted during collection time point, could also indicate the evolution of a host–pathogen relationship in these birds enabling co-infections. However, how these viruses impact the health of these birds can only be speculated at this point, and further pathological studies are required for the complete elucidation of these viruses’ pathogenicity.

Despite the detection and characterisation of 46 parvoviruses and 11 picornaviruses from these samples, there is a high possibility of the presence of many more parvoviruses and picornaviruses due to the detection of partial genome sequences. However, as shown in Figures S1–S5 of the Supplementary Material 2, we can determine the minimum number of chaphamaparvoviruses and picornaviruses infecting the ducks, as the sequences that are being compared are most definitely not identical to each other both at the nucleotide and amino acid level. Also, we cannot completely exclude the minor probability of some of these viruses being from the diet of the bird and not truly from the duck host. It should be noted that using this method, we can detect and characterise other non-avian viruses from the samples as described earlier^6^. Nevertheless, all the virus sequences described here are distantly related and do cluster together along with other avian viruses. Detection of these viruses from different duck samples or culturing and inoculation studies with the viruses could potentially better establish the identity of their hosts.

We found an upstream ORF in some of the picornaviruses and chaphamaparvoviruses in silico; however, at this point, the expression of this ORF is uncertain. The uORF could regulate the translation of the primary ORF and was detected in several viruses such as enteroviruses^38^ and human cytomegalovirus^52^ and shown in silico to be present in some chaphamaparvoviruses^39^. It is previously shown that in enteroviruses, the uORF encode a virus protein that facilitates virus growth in gut epithelial cells, which is the site of initial viral invasion into susceptible hosts^38^. However, further isolation and purification of the uORF protein only could lead to the concrete conclusion on the expression and function of this short peptide in the duck viruses. The presence of other sequence features like the helicase, RdRp, Peptidase C3 and capsid protein domain detected in silico indicate that the virus sequences described here are likely from functional avian viruses. Other virus motifs such as bipartite nuclear localisation signal^53,54^ were also detected in some of the parvoviruses and picornaviruses, although, the detailed analysis on the functions of these motifs and domains exceeds the scope of this paper. It is to be noted that the phospholipase A2 domain was not found in any of the parvoviruses detected from the duck sample, which is consistent with other avian parvoviruses^16^ and avian chaphamaparvovirus^49^.

Finally, as stated above, the avian parvo- and picornaviruses detected and characterised from the bird samples form only a fraction of the total avian virome of these duck species. The identification of 46 different avian parvoviruses and 11 different avian picornaviruses that are distantly related to other currently known viruses from the NCBI dataset indicate the poor understanding of the avian virome and emphasise the need for further elucidation. Further analysis of the NGS reads generated from the wild duck samples is underway to determine the avian virus community of these birds and to determine the factors that influence their ecology.

## Materials and methods

### Sample collection

Fresh wild duck faecal samples were collected from Wallington, south-eastern Victoria, Australia. The samples were collected in late autumn (May 2018), late winter (August 2018) and late spring/early summer (November 2018 and December 2016). Individual Pacific black ducks (PBD), Chestnut teals (CT), Grey teals (GT) and Wood ducks (WD) were able to be captured and sampled as part of this study. Pacific black ducks were captured in May and August 2018 (PBD05.18 and PBD08.18, respectively). No wild Pacific black ducks were captured during November 2018 and consequently, we included pooled juvenile Pacific black duck samples that had been collected in December 2016 (pool PBD12.16 or described as MAD previously^6^) in this study to have samples from three seasonal time points for this duck species. Chestnut teal samples were collected in May, August and November 2018 (CT05.18, CT08.18 and CT11.18). Grey teals were able to be captured and sampled in November 2018 (GT11.18) but at no other time points. Wood ducks were only captured and sampled in August 2018 (WD08.18). All samples were stored at − 80 °C within 1–3 h of collection until processing.

Bird sample collection was approved under Deakin University’s Animal Ethics Committee project number B43-2016 and Department of Environment, Land, Water and Planning permit number 1008206. The current study involving these samples were performed in accordance with relevant guidelines and regulations.

### Virus enrichment from samples

Samples of 3–6 individual ducks were pooled by species and collection date**,** except for the Chestnut teal November 2018 sample where only a single bird was captured at the time of sample collection. Enriching for virus particles followed by nucleic acid extraction was carried out as per a previously optimised protocol in our laboratory^6^. Briefly, the faecal samples were subjected to homogenisation, centrifugation and filtration using a 0.8 µm PES filter. The sample was then divided into two aliquots. Aliquot A was ultracentrifuged, while aliquot B did not undergo ultracentrifugation. Both aliquots were then nuclease treated, followed by nucleic acid extraction with the QIAamp Viral RNA mini kit (Qiagen)^6^.

### Next-generation sequencing (NGS)

The extracted nucleic acids from both aliquot A and B of the samples were subjected to cDNA synthesis and amplification with the SeqPlex RNA Amplification Kit (Sigma) as per the manufacturer’s instructions^6^. Sequencing library preparation was performed using the Ion Fragment Library kit (Life Technologies)^6^. Libraries were quantified and then pooled prior to loading onto Ion 530 or 540 Chips using the Ion Chef Instrument. Following template preparation, the chips were run on an Ion Torrent S5XL System (Thermo Fisher Scientific) as per company protocols. Two individual Chestnut teal samples (CT08.18/11356 and CT08.18/12952) from August 2018 and which formed part of pool CT08.18 were also processed and sequenced as described for the pooled samples, to obtain more virus sequences of a low pathogenicity H9N2 avian influenza virus detected in that sample^4^.

### NGS data analyses

NGS data analyses were carried out as described earlier^6,55,56^. Briefly, all available virus sequences and all RefSeq virus sequences were downloaded from the NCBI GenBank genetic sequence database (Dec 2018), and a local BLAST database created for the two sets of sequences separately. BLASTN query against the two virus reference sequence databases was performed with an e-value cut-off of 1 × 10^−10^ and 1 × 10^–30^. A TBLASTX query against the two virus reference sequence databases was also performed with an e-value cut-off of 1 × 10^−10^. BLAST query results files were converted into spreadsheet files, sorted by virus matches, and a list of potential virus targets created for each sample. The list was then manually inspected to identify viruses of interest. It became apparent that parvoviruses and picornaviruses reads were particularly abundant in many of the samples, and therefore we focused further analyses on these virus families.

The NGS reads were mapped against reference genomes of parvoviruses and picornaviruses identified to be of interest using the TMAP plugin on the Ion Torrent server. The full or partial consensus sequences of the viruses were obtained from TMAP using Integrative Genomics Viewer software (IGV) (Broad Institute, MA, USA) as described earlier^6,55,56^. AssemblerSPAdes 5.6.0 plugin on the Ion Torrent Server was used to generate contigs from the sequence reads of each sample as described earlier^6^. The contigs were queried against the two virus reference sequence databases using BLASTN and TBLASTX to identify virus sequences as described above and using the same e-value cut-offs. Picornavirus and parvovirus contigs of length greater than 500 nucleotides were then used as references in TMAP plugin and trimmed to regions with a mapping quality of 20 or higher and a coverage depth of at least 2 unless specified. Nearly complete sequences for some viruses were also generated by assembling overlapping sequences and by using MEGA7 and magicblast^57^ from contigs and consensus sequences generated from different NGS runs. These sequences were again subjected to TMAP and consensus sequences generated from mapped sequences using IGV to determine the depth of coverage.

The use of the non-ultracentrifuged samples acted as a control to differentiate endogenous sequences compared to sequences coming from exogenous virion. The sequences more abundant in the ultracentrifuged samples and those which did not provide any evidence of host genome sequences attached were considered likely to be from virus genomes.

### ORF prediction and Motif analysis

Open reading frames (ORFs), and features of the virus genomes such as promotors, motifs and nuclear locating signals were identified in silico using Basic Local Alignment Search Tool^58,59^ (BLASTN, BLASTX and BLASTP), NCBI ORFfinder^60^, ScanProsite^61^ and PSORT II^62^. The presence of these features in the expected location in the virus sequences was used as an additional confirmation that reliable consensus sequences had been generated from the reads.

### Phylogenetic analysis of virus sequences

Relevant sequences related to the partial and complete virus consensus sequences identified here were selected from the NCBI GenBank database using BLASTN. Nucleotide and protein sequences were aligned using Clustal-W^63^ using the MEGA 7^64^/MEGACC^65^ software. Maximum Likelihood phylogenetic analyses were conducted using the MEGA 7/MEGACC software by first identifying the best evolutionary model for generating a phylogenetic tree before creating the phylogenetic trees. The robustness of different nodes was assessed by bootstrap analysis using 1,000 replicates for amino acid alignments. The number of nucleotide/amino acid differences between sequences was calculated using the same software.

## Supplementary information

Supplementary information

## Data Availability

All sequences analysed have been deposited in NCBI GenBank. The parvoviruses are under accession numbers MT247729–MT247830, while the picornaviruses are under accession numbers MT247831–MT247856. Other datasets generated or analysed during the current study are available from the corresponding author on reasonable request.
