# Supplementary material for: Metagenomic characterisation of avian parvoviruses and picornaviruses from Australian wild ducks
Source: Sci Rep. 2020 Jul 30;10:12800. doi: 10.1038/s41598-020-69557-z (PMC7393117; doi:10.1038/s41598-020-69557-z)
Supplement: Supplementary file 1 — Supplementary information [file 41598_2020_69557_MOESM1_ESM.pdf]

Supplementary material 1

Metagenomic characterisation of avian parvoviruses and picornaviruses from Australian wild ducks

Running title: Avian parvoviruses and picornaviruses in Australian wild ducks

Supplementary material 1: Details of the sequences of avian parvoviruses and picornaviruses found in the duck samples

Authors:
Jessy Vibin*[1,2], Anthony Chamings[1,2], Marcel Klaassen[3], Tarka Raj Bhatta[1,2], Soren Alexandersen*[1,2,4]
1 Geelong Centre for Emerging Infectious Diseases, Geelong, VIC 3220, Australia;
2 Deakin University, School of Medicine, Geelong, VIC 3220, Australia;
3 Deakin University, Centre for Integrative Ecology, Waurn Ponds, VIC 3216, Australia;
4 Barwon Health, Geelong, VIC 3220, Australia;

*Corresponding Author: jessyatall@gmail.com, soren.alexandersen@deakin.edu.au

Supplementary material 1: Details of the sequences of avian parvoviruses and picornaviruses found in the duck samples

The table provides details of the sequences we provide. The column A gives the virus and the bird from which it was charactertised. It also gives the NCBI accession number of the virus sequence. The column B gives the coverage and mapping quality of the virus consensus sequence. The column C provides the coverage analysis i.e the total number of reads generated for the particular virus sequences at a mapping quality on or above 20 with the minimum aligned length of 100 nucelotides. It also provides the abundance of the virus in the generated NGS data of the sample. The column D provides the protein the virus sequence encodes. The column E-I provides if the protein expressed in a sense or an antisense strand and the start of coding frame position, the nucleotide position of the start of the protein, the nucleotide position of the stop codon/end of the protein and the total length of the ORF. The column J gives the percentage of nucleotide/amino acid identity to its closest relative identified through either MEGA analysis or BLASTP. Finally, the column K provides, if any, features were identified in the sequene using ScanProsite or PSORTII.

	Column A	B	C	D	E	F	G	H	I	J	K
	**Virus | Bird sample | NCBI Accession number**	**Coverage | Mapping quality (at which the final consensus sequences were generated)**	**Coverage analysis (Total no. of reads @ Q20) | Abundance (%)**	**ORF**	**Strand**	**Frame**	**Start**	**Stop**	**Length (nt | aa)**	**Nucleotide/amino acid identity to its closest relative (%)**	**Special features (Promoter / Motifs)**
	**Parvoviruses**										
1	Pacific black duck adeno-associated virus (PBDAAV) | PBD12.16 | MT247729	2-162 (except for nucleotides 1528 to 1637 which was from a single NGS read of Q95) | Q60	549 | 0.007%	NS1	+	2	170	2110	1941 | 646	KX583629: 82.5% in aa (MEGA analysis)	TATA box: 105-110 nt; 2107-2112 nt | Helicase at 309 - 464 aa of NS1 | Bipartite NLS at 153-167aa of the capsid | Arginine rich region profile at 107-150 aa of AAP
				Capsid	+	1	2128	4347	2220 | 739	KX583629: 70.9% in aa (MEGA analysis)	
				Activation Assembly protein (AAP)	+	2	2792	3280	489 | 162		
2	Pacific black duck chaphamaparvovirus 1 (PBDCPaV1) | PBD12.16 | MT247730	25-6079 | Q80	313043 | 4.27%	NS1	+	1	595	2661	2067 | 688	CTCPaV/CT08.18/12952/N39/N40/2864nt: 82.2% in aa (MEGA analysis)	Helicase at 253-438 aa of NS1 | Serine-rich region at 501-560 aa of NS1 | Bipartite NLS at 564-580 aa of NS1
				Capsid	+	2	2609	4228	1620 | 539	CTCPaV/CT08.18/N82/2395nt and CPaV2/CT08.18/12952: 74.5% in aa (MEGA analysis)	
				NP	+	2	1802	2497	696 | 231		
				uORF	+	2	224	694	471 | 156		
3	PBDCPaV/608nt| PBD12.16 | MT247731	4-12 |Q20	45 | 0.02%	608 nt long consensus sequence (partial NS1)	+	1	76	>606	531 | 176	MG846441: 46.3% in aa (blastp)	
4	PBDCPaV/497nt| PBD12.16 | MT247732	3-22 | Q20	2387 | 0.03%	497 nt long consensus sequence (partial NS1)	+	2	<2	>496	495 | 164	HM362436: 54.8% in aa (blastp)	
5	PBDCPaV/1136nt | PBD12.16 | MT247733	6-249 | Q80	1438 | 0.01%	1136 nt long consensus sequence (partial NS1)	+	2	<2	>1135	1134 | 377	CTCPaV2/CT08.18/12952: 90.2% in aa (MEGA analysis)	Helicase at 11-162aa of NS1 | Serine-rich region at 157-257 aa of NS1 | Bipartite NLS at 212-228 aa of NS1
				NP	+	3	444	1040	597 | 198		
6	PBDCPaV/1356nt | PBD12.16 | MT247734	2-512 | Q90	3906 | 0.05%	1356 nt long consensus sequence (partial CP)	+	2	<2	>1354	1353 | 450	CTCPaV2/CT08.18/12952: 96.2% in aa (MEGA analysis)	Glycine-rich region at 129-158 aa of capsid
7	PBDCPaV/957nt | PBD12.16 | MT247735	14-537 | Q90	7858 | 0.10%	957 nt long consensus sequence (partial NS1)	+	2	<2	>955	954 | 317	MG846441: 52.3% in aa (blastp)	
8	PBDCPaV/N104/1995nt | PBD12.16 | MT247736	2-73 | Q80	523 | 0.007%	1995 nt long consensus sequence (partial NS1)	+	1	316	>1995	1680 | 559	CTCPaV1/CT08.18: 83.0% in aa (MEGA analysis)	Helicase at 295-442 aa of NS1 | Serine-rich region at 485-545 aa of NS1
9	PBDCPaV/N104/1295nt | PBD12.16 | MT247737	2-180 | Q80	431 | 0.005%	1295 nt long consensus sequence (partial NS1)	+	1	<1	282	282 | 93	MG846443: 48.4% in aa (blastp)	
				partial CP	+	3	279	>1295	1017 | 338	WDCPaV/N92/N153/1561nt/WD08.18: 94.6% in aa (MEGA analysis)	

10	PBDCPaV/N326/568nt | PBD12.16 | MT247738	5-242 | Q80	1425 | 0.02%	568 nt long consensus sequence (partial NS1)	+	1	<1	>567	567 | 188	MK513532: 37.0% in aa (blastp)	
				partial NP	+	2	<2	472	471 | 156		
11	PBDCPaV/N326/1330nt | PBD12.16 | MT247739	21-956 |Q80	8778 | 0.12%	1330 nt long consensus sequence (partial CP)	+	3	<3	>1328	1326 | 442	CTCPaV2/CT08.18/12952: 95.0% in aa (MEGA analysis)	
12	PBDCPaV/N342/1104nt | PBD12.16 | MT247740	5-90 | Q80	437 | 0.005%	1104 nt long consensus sequence (partial NS1)	+	2	<2	994	993 | 330	CTCPaV/N1466/1009nt/CT08.18: 89.0% in aa (MEGA analysis)	Serine-rich region at 110-230 aa of NS1
				NP	+	3	261	860	600 | 199		
				partial CP	+	1	997	>1104	108 | 35		
13	PBDCPaV/N1301/1107nt | PBD12.16 | MT247741	3-1068 | Q80	9990 | 0.13%	1107 nt long consensus sequence (partial NS1)	+	2	<2	>1105	1104 | 367	CTCPaV3/CT08.18/12952 and CTCPaV/N482/1200nt/CT08.18: 71.7% in aa (MEGA analysis)	
14	PBDCPaV/N521/452nt | PBD12.16 | MT247742	4-27 | Q80	53 | 0.0007%	452 nt long consensus sequence (partial CP)	+	2	<2	>451	450 | 149	MG846443: 49.3% in aa (blastp)	
15	PBDCPaV/N2680/612nt | PBD08.18 | MT247743	2-35 | Q80	60 | 0.0009%	612 nt long consensus sequence (partial NS1)	+	3	<3	437	435 | 144	MG846442: 50.7% in aa (blastp)	
				partial CP	+	2	434	>610	177 | 58		
16	PBDCPaV/N470/2334nt | PBD08.18 | MT247744	2-73 |Q80	824 | 0.01%	2334 nt long consensus sequence (partial NS1)	+	3	<3	1103	1101 | 366	CTCPaV/N39/N40/2864nt/CT08.18/12952: 75.1% in aa (MEGA analysis)	Helicase at 1-120 aa of NS1 | Bipartite NLS at 166-182 aa of NS1
				NP	+	1	256	951	696 | 231		
				partial CP	+	1	1096	>2334	1239 | 412	PBDCPaV1/PBD12.16: 92.4% in aa (MEGA analysis)	
17	PBDCPaV/N1662/589nt | PBD08.18 | MT247745	2-17 | Q80	39 | 0.0005%	589 nt long consensus sequence (partial NS1)	+	1	<1	480	480 | 159	MN175612: 36.2% in aa (blastp)	
				partial CP	+	3	483	>587	105 | 34		
18	PBDCPaV/N1428/1025nt | PBD08.18 | MT247746	2-20 | Q80	50 | 0.0009%	1025 nt long consensus sequence (partial NS1)	+	3	<3	479	477 | 158	MG846443: 53.0% in aa (blastp)	Serine-rich region at 20-51 aa of NS1
				partial NP	+	1	<1	357	357 | 118		
				partial CP	+	1	472	>1023	552 | 183	MG846443: 64.1% in aa (blastp)	
19	PBDCPaV/N1927/394nt | PBD08.18 | MT247747	2-29 | Q80	43 | 0.0006%	394 nt long consensus sequence (partial NS1)	+	2	<2	286	285 | 95	MG846442: 43.4% in aa (blastp)	
				partial NP	+	3	<3	152	150 | 49		
				partial CP	+	1	289	>393	105 | 34		
20	PBDCPaV/N3311/637nt | PBD08.18 | MT247748	2-16 | Q60	35 | 0.0005%	637 nt long consensus sequence (partial CP)	+	2	<2	>637	636 | 211	MG846443: 46.1% in aa (blastp)	
21	PBDCPaV/N1362/474nt | PBD08.18 | MT247749	3-19 | Q80	34 | 0.0005%	474 nt long consensus sequence (partial CP)	+	2	<2	>472	471 | 156	MG846443: 51.0% in aa (blastp)	
22	PBDCPaV/N1168/688nt | PBD08.18 | MT247750	2-16 | Q80	49 | 0.0009%	688 nt long consensus sequence (partial CP)	+	2	<2	>688	687 | 228	MG846443: 42.0% in aa (blastp)	
23	PBDCPaV/N1170/982nt | PBD08.18 | MT247751	2-28 | Q80	222 | 0.003%	982 nt long consensus sequence (partial NS1)	+	2	179	>982	804 | 267	CTCPaV/N39/N40/2864nt/CT08.18/12952: 79.4% in aa (MEGA analysis)	
				partial uORF	+	3	<3	278	276 | 91		
24	PBDCPaV/N141/467nt | PBD08.18 | MT247752	2-22 | Q80	47 | 0.0009%	467 nt long consensus sequence (partial NS1)	+	3	<3	>467	465 | 154	MG846441: 51.2% in aa (blastp)	
25	PBDCPaV/N141/532nt | PBD08.18 | MT247753	12-188 | Q80	464 | 0.007%	532 nt long consensus sequence (partial NS1)	+	3	<3	467	465 | 154	KX272741: 33.3% in aa (blastp)	
				partial NP	+	1	<1	351	351 | 116		

26	PBDCPaV/N141/1311nt | PBD08.18 | MT247754	4-133 | Q80	426 | 0.006%	1311 nt long consensus sequence (partial CP)	+	1	<1	>1311	1311 | 436	CTCPaV3/CT08.18/12952: 85.3% in aa (MEGA analysis)	
27	Pacific balck duck aveparvovirus (PBDAPaV)/N1106/1233nt | PBD08.18 | MT247755	2-176 | Q80	637 | 0.009%	1233 nt long consensus sequence (partial NS1)	+	3	72	>1232	1161 | 386	KC876004: 67.7% in aa (MEGA analysis)	
28	PBDAPaV/N443/650nt | PBD08.18 | MT247756	4-34 | Q80	70 | 0.001%	650 nt long consensus sequence (partial NS1)	+	3	<3	326	324 | 107	KC876004: 67.3% in aa (blastp)	
				hypothetical protein (HP)	+	1	145	321	177 | 58		
29	PBDAPaV/N443/1497nt | PBD08.18 | MT247757	2-112 | Q80	429 | 0.006%	1497 nt long consensus sequence (partial HP)	+	2	<2	472	471 | 156	KC876004: 35.7% in aa (blastp)	Ribosomal protein S14 signature at 83-105 aa of HP
				partial CP	+	3	465	>1496	1032 | 343	KC876004: 69.6% in aa (MEGA analysis)	
30	Chestnut teal chaphamaparvovirus 1 (CTCPaV1) | CT08.18 | MT247758	2-250 | Q90	5483 | 0.02%	NS1	+	3	609	2630	2022 | 673	MG846442 and MG846443: 69.6% in aa (MEGA analysis)	TATA box: 258-263 nt | Helicase at 299-443 aa of NS1 | Serine-rich region at 464-579 aa of NS1
				CP	+	2	2627	4333	1707 | 568	MG846443: 84.8% in aa (MEGA analysis)	
				NP	+	1	1828	2511	684 | 227		
				uORF	+	1	283	726	444 | 147		
31	CTCPaV2 | CT08.18/12952 | MT247759	36-4337 | Q80	3631 | 0.13%	uORF	+	2	254	691	438 | 145		Helicase at 294-445 aa of NS1 | Glycine rich region at 146-175 aa of CP
				NS1	+	1	583	2586	2004 | 667	CTCPaV1/CT08.18: 57.1% in aa (MEGA analysis)	
				CP	+	2	2555	4270	1716 | 571	CTCPaV/CT08.18/N82/N83/2395 nt: 94.5% in aa (MEGA analysis)	
				NP	+	2	1874	2470	597 | 198		
32	CTCPaV3 | CT08.18 /12952 | MT247760	2-1160	7333 | 0.27%	uORF	+	3	252	728	477 | 158		Helicase at 297-441 aa of NS1 | Serine rich region at 498-605 aa of NS1 | Bipartite NLS at 501-517 aa of NS1
				NS1	+	2	614	2641	2028 | 675	CTCPaV1/CT08.18: 60.4% in aa (MEGA analysis)	
				CP	+	1	2644	4326	1683 | 560	CTCPaV2/CT08.18/12952: 76.9% in aa (MEGA analysis)	
				NP	+	3	2064	2516	453 | 150		
33	CTCPaV/N82/N83/1538nt | CT08.18 | MT247761	3-105 | Q80	2022 | 0.01%	1538 nt long consensus sequence (partial NS1)	+	2	611	>1537	927 | 308	CTCPaV2/CT08.18/12952: 94.4% in aa (MEGA analysis)	
				uORF	+	3	282	719	438 | 145		
34	CTCPaV/N82/N83/344nt | CT08.18 | MT247762	2-46 | Q90	461 | 0.002%	344 nt long consensus sequence (partial NS1)	+	2	<2	>343	342 | 113	MN175613: 51.3% in aa (blastp)	
35	CTCPaV/N82/N83/2395nt | CT08.18 | MT247763	2-334 | Q80	5451 | 0.02%	2395 nt long consensus sequence (partial NS1)	+	1	<1	624	624 | 207	MG846442: 37.9% in aa (blastp)	Serine-rich region at 7-94 aa of NS1 | Bipartite NLS at 49-65 aa of NS1
				partial NP	+	2	<2	550	549 | 182		
				CP	+	3	660	2345	1686 | 561	CTCPaV2/CT08.18/12952: 94.4% in aa (MEGA analysis)	
36	CTCPaV/N39/N40/1318nt | CT08.18/12952 | MT247764	3-932 | Q80	2883 | 0.10%	partial CP	+	1	<1	1245	1245 | 414	PBDCPaV1/PBD12.16: 87.4% in aa (MEGA analysis)	
37	CTCPaV/N39/N40/2864nt | CT08.18/12952 | MT247765	4-864 | Q80	6766 | 0.25%	NS1	+	1	580	2610	2031 | 676	PBD12.16/PBDCPaV1: 81.9% in aa (MEGA analysis)	Helicase at 253-438 aa of NS1 | Serine rich region at 501-534 aa of NS1 | Zinc finger C2H2 type domain at 85-108 aa of NS1 | Bipartite NLS at 487-503 of NS1
				partial CP	+	2	2567	>2863	297 | 98		
				NP	+	2	1787	2473	687 | 228		

38	CTCPaV/N158/N481/764nt | CT08.18 | MT247766	2-207 | Q80	1187 | 0.006%	764 nt long consensus sequence (partial NS1)	+	3	<3	>764	762 | 253	KM254174: 49.2% in aa (blastp)	
39	CTCPaV/N158/707nt | CT08.18 | MT247767	4-121 | Q80	533 | 0.002%	707 nt long consensus sequence (partial NS1)	+	2	590	>706	117 | 38		
				uORF	+	3	243	689	447 | 148		
40	CTCPaV/N158/N481/707nt | CT08.18 | MT247768	2-73 | Q80	157 | 0.004%	707 nt long consensus sequence (partial NS1)	+	3	<3	>707	705 | 234	MG846441: 44.6% in aa (blastp)	
				partial NP	+	1	283	>705	423 | 140		
41	CTCPaV/N752/444nt | CT08.18 | MT247769	2-53 | Q80	168 0.0005%	444 nt long consensus sequence (partial CP)	+	2	<2	>442	441 | 146	MG846442: 51.8% in aa (blastp)	
42	CTCPaV/N564/1347nt | CT08.18 | MT247770	2-140 | Q80	994 | 0.003%	1347 nt long consensus sequence (partial CP)	+	1	<1	>1347	1347 | 448	GTCPaV/N32/N66/1153nt/GT11.18: 89.9% in aa (MEGA analysis)	
43	CTCPaV/N482/733nt | CT08.18 | MT247771	4-39 | Q80	174 | 0.0009%	733 nt long consensus sequence (partial NS1)	+	2	<2	>733	732 | 243	MG846443: 39.9% in aa (blastp)	Serine-rich region at 77-189 aa of NS1 | Neutral zinc metallopeptidases at 189-198 aa of NS1
				NP	+	3	198	638	441 | 146		
44	CTCPaV/N482/1200nt | CT08.18 | MT247772	3-204 | Q80	1317 | 0.007%	1200 nt long consensus sequence (partial NS1)	+	1	340	>1200	861 | 286	CTCPaV3/CT08.18/12952: 96.1% in aa (MEGA analysis)	
				partial uORF	+	2	<2	454	453 | 150		
45	CTCPaV/N1262/N736/1339nt | CT08.18 | MT247773	2-98 | Q80	890 | 0.002%	1339 nt long consensus sequence (partial CP)	+	1	<1	>1338	1338 | 446	GTCPaV/N34/1114nt/GT11.18: 95.7% in aa (MEGA analysis)	
46	CTCPaV/N559/N1308/1138nt | CT08.18 | MT247774	3-48 | Q80	310 | 0.001%	1138 nt long consensus sequence (partial NS1)	+	2	<2	>1138	1137 | 378	CTCPaV3/CT08.18/12952: 99.4% in aa (MEGA analysis)	Helicase at 9-153 aa aa of NS1 | Serine-rich region at 210-317 aa of NS1 | Bipartite NLS at 213-229 aa of NS1
				NP	+	3	588	1040	453 | 150		
47	CTCPaV/N1992/N5037/608nt | CT08.18 | MT247775	5-32 | Q80	212 | 0.001%	608 nt long consensus sequence (partial CP)	+	2	<2	>607	606 | 201	MG846443: 54.7% in aa (blastp)	
48	CTCPaV/N7198/793nt | CT08.18 | MT247776	4-51 | Q80	478 | 0.002%	793 nt long consensus sequence (partial NS1)	+	2	<2	712	711 | 236	MG846443: 43.8% in aa (blastp)	Serine-rich region at 6-111 aa of NS1
				partial NP	+	3	<3	596	594 | 197		
				partial CP	+	3	714	>791	78 | 25		
49	CTCPaV/N4625/548nt | CT08.18 | MT247777	2-111 | Q80	258 | 0.001%	548 nt long consensus sequence (partial NS1)	+	2	<2	>547	546 | 181	MG846442: 49.7% in aa (blastp)	
50	CTCPaV/N3159/804nt | CT08.18 | MT247778	2-29 | Q80	207 | 0.0006%	804 nt long consensus sequence (partial CP)	+	3	<3	>803	801 | 266	MG846443: 40.0% in aa (blastp)	
51	CTCPaV/N3813/1032nt | CT08.18 | MT247779	2-18 | Q80	274 | 0.001%	1032 nt long consensus sequence (partial NS1)	+	2	677	>1030	354 | 117	MG846442: 45.7% in aa (blastp)	TATA box: 216-221 nt
				uORF	+	3	243	677	435 | 144		
52	CTCPaV/N3269/492nt | CT08.18 | MT247780	3-32 | Q80	81 | 0.0006%	492 nt long consensus sequence (partial CP)	+	1	<1	>492	492 | 163	MG846443: 51.1% in aa (blastp)	
53	CTCPaV/N1115/1024nt | CT08.18 | MT247781	2-53 | Q80	403 | 0.002%	1024 nt long consensus sequence (partial NS1)	+	3	381	>1022	642 | 213	MG846441: 48.8% in aa (blastp)	
				uORF	+	1	55	492	438 | 145		
54	CTCPaV/N1148/1113nt | CT08.18 | MT247782	3-54 | Q80	269 | 0.0008%	1113 nt long consensus sequence (partial NS1)	+	1	<1	>1113	1113 | 370	KM254174: 49.4% in aa (blastp)	
55	CTCPaV/N1466/1009nt | CT08.18 | MT247783	2-85 | Q80	395 | 0.002%	1009 nt long consensus sequence (partial NS1)	+	2	<2	901	900 | 299	PBDCPaV/N342/1104nt/PBD12.16: 87.9% in aa (MEGA analysis)	Serine-rich region at 79-199 aa of NS1
				NP	+	3	117	767	651 | 216		
				partial CP	+	1	904	>1008	105 | 34		

56	CTCPaV/N481/881nt | CT08.18/11356 | MT247784	31-581 | Q80	169 | 0.004%	881 nt long consensus sequence (partial NS1)	+	1	<1	321	321 | 106	MG846443: 31.3% in aa (blastp)	
				partial CP	+	2	269	>550	282 | 93		
				partial NP	+	2	<2	157	156 | 51		
57	CTCPaV/N194/N189/1197nt | CT08.18/12952 | MT247785	4-152 | Q80	362 | 0.013%	1197 nt long consensus sequence (partial NS1)	+	2	<2	871	870 | 289	CTCPaV/N66/1550nt/CT08.18/12952: 83.3% in aa (MEGA analysis)	Serine rich region at 69-197 aa of NS1
				partial CP	+	1	871	>1197	327 | 108		
				NP	+	3	150	737	588 | 195		
58	CTCPaV/N194/N189/603nt | CT08.18/12952 | MT247786	4-99 | Q80	203 | 0.005%	603 nt long consensus sequence (partial NS1)	+	2	<2	>601	600 | 199	MG846441: 44.5% in aa (blastp)	
59	CTCPaV/N238/595nt | CT08.18/12952 | MT247787	2-70 | Q80	175 | 0.006%	595 nt long consensus sequence (partial NS1)	+	2	<2	>595	594 | 197	MG846441: 53.0% in aa (blastp)	
60	CTCPaV/N589/843nt | CT08.18/12952 | MT247788	6-152 | Q80	479 | 0.017%	843 nt long consensus sequence (partial NS1)	+	2	<2	445	444 | 147	MG846442: 37.7% in aa (blastp)	
				partial CP	+	1	448	>843	396 | 131	MG846443: 60.1% in aa (blastp)	
				partial NP	+	3	<3	311	309 | 102		
61	CTCPaV/N691/612nt | CT08.18/12952 | MT247789	2-100 | Q80	273 | 0.01%	612 nt long consensus sequence (partial CP)	+	3	<3	>611	609 | 202	MG846443: 53.2% in aa (blastp)	
62	CTCPaV/N926/670nt | CT08.18/11356 | MT247790	2-427 | Q80	1226 | 0.006%	670 nt long consensus sequence (partial CP)	+	2	<2	>670	669 | 222	KY312549: 43.9% in aa (blastp)	
63	CTCPaV/N2762/685nt | CT08.18/12952 | MT247791	2-44 | Q80	84 | 0.003%	685 nt long consensus sequence (partial NS1)	+	1	<1	525	525 | 174		
				partial CP	+	2	473	>685	213 | 70	KY312549: 50.0% in aa (blastp)	
				partial NP	+	2	<2	361	360 | 119	MN175613: 34.4% in aa (blastp)	
64	CTCPaV/N353/738nt | CT08.18/12952 | MT247792	2-22 | Q80	37 | 0.001%	738 nt long consensus sequence (partial NS1)	+	1	<1	>738	738 | 245	MG846443: 40.8% in aa (blastp)	
				partial NP	+	2	374	>736	363 | 120		
65	CTCPaV/N15/1244nt | CT08.18/12952 | MT247793	2-281 | Q80	573 | 0.021%	1244 nt long consensus sequence (partial NS1)	+	1	<1	990	990 | 329	GTCPaV/N44/1018nt/GT11.18: 79.9% in aa (MEGA analysis)	Serine rich region at 140-215 aa of NS1
				partial CP	+	3	990	>1244	255 | 84	MK988620: 51.9% in aa (blastp)	
				NP	+	2	254	874	621 | 206		
66	CTCPaV/N15/856nt | CT08.18/12952 | MT247794	2-659 | Q80	932 | 0.03%	856 nt long consensus sequence (partial CP)	+	3	<3	>854	852 | 283	MG846443: 41.6% in aa (blastp)	
67	CTCPaV/N1167/836nt | CT08.18/12952 | MT247795	2-121 | Q80	219 | 0.008%	836 nt long consensus sequence (partial NS1)	+	3	258	>836	579 | 192	MG846441: 55.7% in aa (blastp)	
68	CTCPaV/N96/1378nt | CT08.18/12952 | MT247796	2-130 | Q80	421 | 0.015%	1378 nt long consensus sequence (partial NS1)	+	2	<2	1051	1050 | 349	WDCPaV/N92/N153/1351nt/WD08.18: 78.5% in aa (MEGA analysis)	
				partial CP	+	1	1051	>1377	327 | 108	MG846443: 60.1% in aa (blastp)	
				NP	+	3	276	929	654 | 217		
69	CTCPaV/N71/1371nt | CT08.18/12952 | MT247797	2-79 | Q80	329 | 0.01%	1371 nt long consensus sequence (partial NS1)	+	1	<1	1176	1176 | 391	PBDCPaV1/PBD12.16: 85.9% in aa (MEGA analysis)	Helicase at 1-142 aa of NS1 | Serine rich region at 158-265 aa of NS1
				partial CP	+	2	1127	>1369	243 | 80	MN175613: 54.4% in aa (blastp)	
				NP	+	2	320	1015	696 | 231		

70	CTCPaV/N602/1128nt | CT08.18/12952 | MT247798	2-108 | Q80	252 | 0.009%	1128 nt long consensus sequence (partial CP)	+	2	<2	>1126	1125 | 374	GTCPaV/N34/1114nt/GT11.18: 96.8% in aa (MEGA analysis)	
71	CTCPaV/N66/1550nt | CT08.18/12952 | MT247799	2-45 | Q80	205 | 0.007%	1550 nt long consensus sequence (partial NS1)	+	2	<2	874	873 | 290	CTCPaV/N194/N189/1197nt/CT08.18/12952: 83.4% in aa (MEGA analysis)	Serine rich region at 70-200 aa of NS1
				partial CP	+	1	874	>1548	675 | 224	MG846443: 52.4% in aa (blastp)	
72	CTCPaV/N175/754nt | CT08.18/12952 | MT247800	2-24 | Q80	109 | 0.004%	754 nt long consensus sequence (partial NS1)	+	2	107	>754	648 | 215	MG846441: 46.3% in aa (blastp)	
73	CTCPaV/N34/1739nt | CT08.18/12952 | MT247801	2-214 | Q80	650 | 0.02%	1739 nt long consensus sequence (partial NS1)	+	1	<1	756	756 | 251	WDCPaV/N59/1200nt/WD08.18: 77.6% in aa (MEGA analysis)	Serine rich region at 100-165 aa of NS1
				partial CP	+	3	759	>1739	981 | 326	CTCPaV3/CT08.18/12952: 81.9% in aa (MEGA analysis)	
				NP	+	2	23	637	615 | 204		
74	CTCPaV/N695/944nt | CT08.18/11356 | MT247802	2-30 | Q80	74 | 0.001%	944 nt long consensus sequence (partial NS1)	+	3	<3	779	777 | 258	WDCPaV/N59/1200nt/WD08.18: 70.1% in aa (MEGA analysis)	Serine rich region at 47-181 aa of NS1
				partial CP	+	2	782	>943	162 | 53		
				NP	+	1	58	660	603 | 200		
75	CTCPaV/N558/941nt | CT11.18 | MT247803	2-12 | Q60	87 | 0.004%	941 nt long consensus sequence (partial NS1)	+	1	340	>939	600 | 199	MG846443: 47.7% in aa (blastp)	
76	CTCPaV/N326/N289/637nt | CT11.18 | MT247804	2-27 | Q60	124 | 0.006%	637 nt long consensus sequence (partial NS1)	+	3	<3	>635	633 | 210	KM254174: 38.3% in aa (blastp)	Bipartite NLS at 86-102 aa of NS1
				NP	+	1	58	360	303 | 100		
77	CTCPaV/N326/499nt | CT11.18 | MT247805	2-33 | Q60	59 | 0.002%	499 nt long consensus sequence (partial CP)	+	2	<2	>499	498 | 165	MG846443: 48.5% in aa (blastp)	
78	CTCPaV/N3223/379nt | CT11.18 | MT247806	2-29 | Q60	57 | 0.002%	379 nt long consensus sequence (partial CP)	+	2	<2	>379	378 | 125	MG846442: 51.1% in aa (blastp)	
79	CTCPaV/N2203/N1060/767nt | CT11.18 | MT247807	3-31 | Q80	112 | 0.005%	767 nt long consensus sequence (partial NS1)	+	3	<3	716	714 | 237	MG846443: 37.1% in aa (blastp)	Serine rich region at 22-186 aa of NS1
				partail NP	+	1	<1	597	597 | 198		
80	CTCPaV/N735/N583/578nt | CT11.18 | MT247808	2-23 | Q60	66 | 0.003%	578 nt long consensus sequence (partial NS1)	+	3	<3	>578	576 | 191	MG846443: 52.5% in aa (blastp)	
81	Wood duck chaphamaparvovirus (WDCPaV)/N19/1003nt | WD08.18 | MT247809	5-277 | Q80	1262 | 0.01%	1003 nt long consensus sequence (partial NS1)	+	3	459	>1001	543 | 180	MG846441: 49.7% in aa (blastp)	
				partial uORF	+	1	<1	459	459 | 152		
82	WDCPaV/N19/2024nt | WD08.18 | MT247810	2-330 | Q80	3954 | 0.05%	2024 nt long consensus sequence (partial NS1)	+	1	<1	1335	1335 | 444	CTCPaV1/CT08.18: 72.7% in aa (MEGA analysis)	Helicase at 66-211 aa aa of NS1 | Serine-rich region at 215-316 aa of NS1
				NP	+	2	551	1201	651 | 216		
				partial CP	+	3	1335	>2024	690 | 229	MG846443: 55.6% in aa (blastp)	
83	WDCPaV/N19/992nt | WD08.18 | MT247811	2-83 | Q80	579 | 0.008%	992 nt long consensus sequence (partial CP)	+	1	<1	960	960 | 319	MG846443: 45.8% in aa (blastp)	
84	WDCPaV/N59/543nt | WD08.18 | MT247812	2-55 | Q80	186 | 0.002%	543 nt long consensus sequence (partial NS1)	+	2	<2	>541	540 | 179	MG846442: 50.0% in aa (blastp)	
85	WDCPaV/N59/1200nt | WD08.18 | MT247813	3-124 | Q80	843 | 0.005%	1200 nt long consensus sequence (partial NS1)	+	3	<3	959	957 | 318	CTCPaV/N34/1739nt/CT08.18/12952: 82.3% in aa (MEGA analysis)	Serine-rich region at 135-232 aa of NS1 | Arginine rich region at 145-171 aa of NS1
				NP	+	1	220	840	621 | 206		
				partial CP	+	2	959	>1198	240 | 79	KY312549: 48.7% in aa (blastp)	

<table>
<tr><td rowspan="2">86</td><td rowspan="2">WDCPaV/N120/272nt | WD08.18 | MT247814</td><td rowspan="2">3-30 | Q80</td><td rowspan="2">45 | 0.0005%</td><td>272 nt long consensus sequence (partial NS1)</td><td>+</td><td>2</td><td><2</td><td>>271</td><td>270 | 89</td><td>MG846443: 45.5% in aa (blastp)</td><td rowspan="2"></td></tr>
<tr><td>partial NP</td><td>+</td><td>3</td><td><3</td><td>>272</td><td>270 | 89</td><td></td></tr>
<tr><td rowspan="3">87</td><td rowspan="3">WDCPaV/N120/866nt | WD08.18 | MT247815</td><td rowspan="3">2-51 | Q80</td><td rowspan="3">227 | 0.003%</td><td>866 nt long consensus sequence (partial NS1)</td><td>+</td><td>1</td><td><1</td><td>441</td><td>441 | 146</td><td>MG846443: 41.7% in aa (blastp)</td><td rowspan="3"></td></tr>
<tr><td>partial NP</td><td>+</td><td>2</td><td><2</td><td>316</td><td>315 | 104</td><td></td></tr>
<tr><td>partial CP</td><td>+</td><td>3</td><td>441</td><td>>866</td><td>426 | 141</td><td>MG846443: 61.5% in aa (blastp)</td></tr>
<tr><td>88</td><td>WDCPaV/N120/1216nt | WD08.18 | MT247816</td><td>3-43 | Q80</td><td>412 | 0.002%</td><td>1216 nt long consensus sequence (partial CP)</td><td>+</td><td>2</td><td><2</td><td>1180</td><td>1179 | 392</td><td>WDCPaV/N92/N153/1561nt/WD08.18: 83.1% in aa (MEGA analysis)</td><td></td></tr>
<tr><td rowspan="2">89</td><td rowspan="2">WDCPaV/N92/N153/845nt | WD08.18 | MT247817</td><td rowspan="2">2-228 | Q80</td><td rowspan="2">1474 | 0.01%</td><td>845 nt long consensus sequence (partial NS1)</td><td>+</td><td>1</td><td>256</td><td>>843</td><td>588 | 195</td><td>MG846441: 54.7% in aa (blastp)</td><td rowspan="2"></td></tr>
<tr><td>partial uORF</td><td>+</td><td>2</td><td><2</td><td>367</td><td>366 | 121</td><td></td></tr>
<tr><td rowspan="3">90</td><td rowspan="3">WDCPaV/N92/N153/1351nt | WD08.18 | MT247818</td><td rowspan="3">2-376 | Q80</td><td rowspan="3">5392 | 0.02%</td><td>1351 nt long consensus sequence (partial NS1)</td><td>+</td><td>3</td><td><3</td><td>1295</td><td>1293 | 430</td><td>CTCPaV1/CT08.18: 77.2% in aa(MEGA analysis)</td><td rowspan="3">Helicase at 53-200 aa aa of NS1 | Serine-rich region at 202-342 aa of NS1</td></tr>
<tr><td>NP</td><td>+</td><td>1</td><td>580</td><td>1176</td><td>597 | 198</td><td></td></tr>
<tr><td>partial CP</td><td>+</td><td>2</td><td>1292</td><td>>1351</td><td>60 | 20</td><td></td></tr>
<tr><td>91</td><td>WDCPaV/N92/N153/1561nt | WD08.18 | MT247819</td><td>5-65 | Q80</td><td>1722 | 0.01%</td><td>1561 nt long consensus sequence (partial CP)</td><td>+</td><td>1</td><td><1</td><td>1539</td><td>1539 | 512</td><td>PBDCPaV/N104/1295nt/PBD12.16: 96.4% in aa (MEGA analysis)</td><td></td></tr>
<tr><td rowspan="2">92</td><td rowspan="2">WDCPaV/N85/N87/1009nt | WD08.18 | MT247820</td><td rowspan="2">2-100 | Q80</td><td rowspan="2">358 | 0.006%</td><td>1009 nt long consensus sequence (partial NS1)</td><td>+</td><td>1</td><td>421</td><td>>1008</td><td>588 | 195</td><td>MG846443: 60.2% in aa (blastp)</td><td rowspan="2"></td></tr>
<tr><td>uORF</td><td>+</td><td>2</td><td>95</td><td>541</td><td>447 | 148</td><td></td></tr>
<tr><td>93</td><td>WDCPaV/N85/N87/510nt | WD08.18 | MT247821</td><td>2-88 | Q80</td><td>316 | 0.003%</td><td>510 nt long consensus sequence (partial NS1)</td><td>+</td><td>1</td><td><1</td><td>>510</td><td>510 | 169</td><td>ADZ48579: 80.5% in aa (blastp)</td><td></td></tr>
<tr><td rowspan="3">94</td><td rowspan="3">WDCPaV/N85/N87/1736nt | WD08.18 | MT247822</td><td rowspan="3">3-420 | Q80</td><td rowspan="3">1696 | 0.02%</td><td>1736 nt long consensus sequence (partial NS1)</td><td>+</td><td>1</td><td><1</td><td>777</td><td>777 | 258</td><td>CTCPaV1/CT08.18: 70.9% in aa (MEGA analysis)</td><td rowspan="3"></td></tr>
<tr><td>NP</td><td>+</td><td>2</td><td>209</td><td>655</td><td>447 | 148</td><td></td></tr>
<tr><td>partial CP</td><td>+</td><td>3</td><td>774</td><td>>1736</td><td>963 | 320</td><td>CTCPaV1/CT08.18: 89.0% in aa (MEGA analysis)</td></tr>
<tr><td rowspan="2">95</td><td rowspan="2">WDCPaV/N26/606nt | WD08.18 | MT247823</td><td rowspan="2">5-108 | Q80</td><td rowspan="2">333 | 0.004%</td><td>606 nt long consensus sequence (partial NS1)</td><td>+</td><td>1</td><td>478</td><td>>606</td><td>129 | 42</td><td></td><td rowspan="2">TATA box: 129-134 nt</td></tr>
<tr><td>uORF</td><td>+</td><td>2</td><td>152</td><td>589</td><td>438 | 145</td><td></td></tr>
<tr><td>96</td><td>WDCPaV/N26/655nt | WD08.18 | MT247824</td><td>5-42 | Q80</td><td>139 | 0.001%</td><td>655 nt long consensus sequence (partial NS1)</td><td>+</td><td>3</td><td><3</td><td>>653</td><td>651 | 216</td><td>MG846441: 48.8% in aa (blastp)</td><td>C-type lectin domain at 98-124 aa of NS1</td></tr>
<tr><td rowspan="2">97</td><td rowspan="2">WDCPaV/N26/699nt | WD08.18 | MT247825</td><td rowspan="2">6-132 | Q80</td><td rowspan="2">673 | 0.008%</td><td>699 nt long consensus sequence (partial NS1)</td><td>+</td><td>1</td><td><1</td><td>693</td><td>693 | 230</td><td>MG846443: 39.6% in aa (blastp)</td><td rowspan="2">Serine-rich region at 2-163 aa of NS1</td></tr>
<tr><td>partial NP</td><td>+</td><td>2</td><td><2</td><td>580</td><td>579 | 192</td><td></td></tr>
<tr><td>98</td><td>WDCPaV/N26/1236nt | WD08.18 | MT247826</td><td>2-199 | Q80</td><td>711 | 0.01%</td><td>1236 nt long consensus sequence (partial CP)</td><td>+</td><td>1</td><td><1</td><td>>1236</td><td>1236 | 411</td><td>CTCPaV/N602/1128nt/CT08.18/12952 and GTCPaV/N34/1114nt/GT11.18: 89.8% in aa (MEGA analysis)</td><td></td></tr>
<tr><td>99</td><td>Grey teal chaphamaparvovirus (GTCPaV)/N29/695nt | GT11.18 | MT247827</td><td>2-30 | Q80</td><td>74 | 0.005%</td><td>695 nt long consensus sequence (partial NS1)</td><td>+</td><td>3</td><td>246</td><td>>695</td><td>450 | 149</td><td>MG846441: 46.4% in aa (blastp)</td><td></td></tr>
<tr><td rowspan="2">100</td><td rowspan="2">GTCPaV/N44/1018nt | GT11.18 | MT247828</td><td rowspan="2">2-61 | Q80</td><td rowspan="2">177 | 0.01%</td><td>1018 nt long consensus sequence (partial NS1)</td><td>+</td><td>1</td><td><1</td><td>954</td><td>954 | 317</td><td>CTCPaV/N15/1244nt/CT08.18/12952: 79.1% in aa (MEGA analysis)</td><td rowspan="2">Serine rich region at 107-192 aa of NS1</td></tr>
<tr><td>NP</td><td>+</td><td>2</td><td>230</td><td>838</td><td>609 | 202</td><td></td></tr>
</table>

101	GTCPaV/N32/N66/1153nt | GT11.18 | MT247829	2-36 | Q80	222 | 0.01%	1153 nt long consensus sequence (partial CP)	+	2	<2	>1153	1152 | 383	CTCPaV/N564/1347nt/CT08.18: 88.2% in aa (MEGA analysis)	
102	GTCPaV/N34/1114nt | GT11.18 | MT247830	2-37 | Q60	141 | 0.009%	1114 nt long consensus sequence (partial CP)	+	2	<2	>1114	1113 | 370	CTCPaV/N602/1128nt/CT08.18/12952: 96.7% in aa (MEGA analysis)	Threonine rich region at 318-364 aa of CP
	**Picornaviruses**										
1	Chestnut teal aalivirus (CTAaV)/N155/242nt | CT05.18 | MT247831	15-78 | Q80	167 | 0.001%	242 nt long consensus sequence (partial PP)	+	2	<2	>241	240 | 79	KJ000696.1: 61.3% in aa (blastp)	
2	CTAaV/N155/1084nt | CT05.18 | MT247832	5-78 | Q80	395 | 0.002%	1084 nt long consensus sequence (partial PP)	+	2	<2	>1084	1083 | 360	PBDAaV/4768nt/PBD05.18: 62.3% in aa (MEGA analysis)	PiV capsid protein domain: 1-119 and 203-347 aa of PP
3	CTAaV/N351/654nt | CT05.18 | MT247833	2-211 | Q80	616 | 0.004%	654 nt long consensus sequence (partial PP)	+	2	<2	>652	652 | 216	KJ000696.1: 46.4% in aa (blastp)	
4	CTAaV/N145/2010nt | CT05.18 | MT247834	2-353 | Q80	1540 | 0.01%	2010 nt long consensus sequence (partial PP)	+	3	<3	>2009	2007 | 668	KJ000696.1: 49.84% in aa (blastp)	
5	CTAaV/N55/2515nt | CT05.18 | MT247835	3-301 | Q80	1426 | 0.009%	2515 nt long consensus sequence (partial PP)	+	1	<1	>2514	2514 | 837	KJ000696.1: 65.5% in aa (MEGA analysis)	RdRp: 491-832 aa of PP; Peptidase C3: 290-476 aa of PP; RNA helicase: 1-125 aa of PP
6	Chestnut teal megrivirus (CTMeV)/N18839/583nt | CT08.18 | MT247836	2-8 | Q80	29 | 0.00008%	583 nt long consensus sequence (partial PP)	+	3	<3	>581	579 | 192	MF405436.1: 53.33% in aa (blastp)	PiV capsid protein domain: 1-176 aa of PP
7	CTMeV/N25167/363nt | CT08.18 | MT247837	2-8 | Q80	14 | 0.0001%	363 nt long consensus sequence (partial PP)	+	3	141	>362	222 | 73	MK204391: 72.6% in aa (blastp)	
8	Chestnut teal sicinivirus-like virus (CTSV-like virus)/505nt | CT08.18 | MT247838	3-9 | Q80	32 | 0.00009%	505 nt long consensus sequence (partial PP)	+	3	<3	>503	501 | 166	MG846476.1: 49.7% in aa (blastp)	
9	Pacific black duck sicinivirus-like virus (PBDSV-like virus)/4111nt | PBD05.18 | MT247839	2-18600 | Q80	62948 | 1.55%	4111 nt long consensus sequence (partial PP)	+	1	1051	>4110	3060 | 1020	MG846480.1 and MG846481.1: 63.0% in aa (MEGA analysis)	PiV capsid protein domain: 638-798 and 851-1013 aa of PP
				uORF	+	3	900	1319	420 | 139		
10	PBDSV-like virus/4792nt | PBD05.18 | MT247840	18-7269 | Q80	51517 | 1.27%	4792 nt long consensus sequence (partial PP)	+	1	<1	4488	4488 | 1495	KF979331.1: 61.4% in aa (MEGA analysis)	RdRp: 1034-1489 aa of PP; Peptidase C3: 841-997 aa of PP; RNA helicase: 454-622 aa of PP; Bipartite NLS: 801-817
11	Pacific black duck aalivirus (PBDAaV)/4768nt | PBD05.18 | MT247841	2-666 | Q80	7396 | 0.18%	4768 nt long consensus sequence (partial PP)	+	3	573	>4766	4194 | 1397	KJ000696.1: 92.4% in aa (MEGA analysis)	PiV capsid protein domain: 74-262 and 336-492 aa of PP
				uORF	+	1	406	603	198 | 65		
12	PBDAaV/4182nt | PBD05.18 | MT247842	1-330 | Q80	4523 | 0.11%	4182 nt long consensus sequence (partial PP)	+	1	<1	3858	3858 | 1285	KJ000696.1: 98.8% in aa (MEGA analysis)	RdRp: 844-1266 aa of PP; Peptidase C3: 646-829 aa of PP; RNA Helicase: 314-479 aa of PP; AAA+ superfamily: 330-466 aa of PP

13	Pacific black duck anativirus like virus (PBDAnV-like virus)/5691nt | PBD08.18 | MT247843	2-338 | Q90	3037 | 0.02%	5691 nt long consensus sequence (partial PP)	+	2	<2	>5689	5688 | 1896	AY563023.1: 52.8% in aa (MEGA analysis)	RdRp: 1550-1896 aa of PP; Peptidase C3: 1358-1533 aa of PP; RNA Helicase: 1014-1174 aa of PP; Picornavirus 2B protein: 815-898 aa of PP; PiV capsid protein domain: 57-239, 304-466 and 570-767 aa of PP
14	PBDAaV/7615nt | PBD08.18 | MT247844	2-295 | Q90	7138 | 0.06%	7615 nt long consensus sequence (partial PP)	+	2	<2	>7615	7614 | 2538	KJ000696.1: 82.8% in aa (MEGA analysis)	RdRp: 2107-2529 aa of PP; Peptidase C3: 1909-2092 aa of PP; RNA Helicase: 1581-1743 aa of PP; AAA+ superfamily: 1596-1732 aa of PP; PiV capsid protein domain: 2-133 and 197-361 aa of PP
15	Pacific black duck megrivirus (PBDMeV)/3747nt | PBD08.18 | MT247845	19-631 | Q90	10011 | 0.19%	3747 nt long consensus sequence (partial PP)	+	3	594	>3746	3153 | 1050	MK204391.1: 98.7% in aa (MEGA analysis)	PiV capsid protein domain: 155-312, 379-584 and 620-811 aa of PP
16	PBDMeV/455nt | PBD08.18 | MT247846	2-23 | Q90	71 | 0.001%	455 nt long consensus sequence (partial PP)	+	3	<3	>455	453 | 150	MK204391.1: 97.3% in aa (blastp)	
17	PBDMeV/876nt | PBD08.18 | MT247847	3-102 | Q90	461 | 0.008%	876 nt long consensus sequence (partial PP)	+	2	<2	>874	873 | 290	MK204391.1: 98.9% in aa (blastp)	
18	PBDMeV/1572nt | PBD08.18 | MT247848	5-194 | Q90	1446 | 0.02%	1572 nt long consensus sequence (partial PP)	+	3	<3	>1571	1569 | 522	MK204417.1: 80.0% in aa (blastp)	RNA helicase: 192-357 aa of PP
19	PBDMeV/2821nt | PBD08.18 | MT247849	2-1565 | Q90	11763 | 0.22%	2821 nt long consensus sequence (partial PP)	+	3	<3	2402	2400 | 799	MK204391.1: 99.2% in aa (MEGA analysis)	RdRp: 339-799 aa of PP; Peptidase C3: 123-320 aa of PP
20	Wood duck megrivirus (WDMeV)/6497nt | WD08.18 | MT247850	2-7291 | Q80	487500 | 6.12%	6497 nt long consensus sequence (partial PP)	+	2	602	>6496	5895 | 1964	KY369300.1: 95.0% in aa (MEGA anaylsis)	PiV capsid protein domain: 155-304, 370-570 and 607-802 aa of PP; RNA helicase: 1667-1832 aa of PP; bipartite NLS: at 1325-1341
				uORF	+	1	373	591	219 | 72		
21	WDMeV/N9/2933nt | WD08.18 | MT247851	2-4472 | Q80	277227 | 3.48%	2933 nt long consensus sequence (partial PP)	+	3	<3	2597	2595 | 864	WDMeV/4874nt/WD08.18: 99.5 % in aa (MEGA analysis)	RdRp: 404-864 aa of PP; Peptidase C3: 189-385 aa of PP
22	WDMeV/N87/2221nt | WD08.18 | MT247852	2-309 | Q80	198891 | 2.5%	2221 nt long consensus sequence (partial PP)	+	2	<2	>2221	2220 | 739	WDMeV/4874nt/WD08.18: 96.8 % in aa (MEGA analysis)	RdRp: 374-703 aa of PP; Peptidase C3: 159-355 aa of PP
23	WDMeV/N292/1296nt | WD08.18 | MT247853	5-197 | Q80	775 | 0.009%	1296 nt long consensus sequence (partial PP)	+	1	<1	>1296	1296 | 431	KY369300.1: 57.4% in aa (MEGA anaylsis)	PiV capsid protein domain: 115-261 and 331-421 aa of PP
24	WDMeV/N12/4874nt | WD08.18 | MT247854	2-332 | Q80	243566 | 3.06%	4874 nt long consensus sequence (partial PP)	+	3	<3	4826	4824 | 1607	WDMeV/2933nt/WD08.18: 99.7% in aa (MEGA analysis)	RdRp: 1147-1607 aa of PP; Peptidase C3: 932-1128 aa of PP; RNA helicase: 485-650 aa of PP; Bipartite NLS: 143-159

25	WDMeV/N281/622nt | WD08.18 | MT247855	3-123 | Q80	19643 | 0.24%	622 nt long consensus sequence (partial PP)	+	3	<3	>620	618 | 205	WDMeV/6497nt/WD08.18: 98.1% in aa (MEGA analysis)	Malic enzyme, NAD binding domain: 85-128 aa of PP; Bipartite NLS: 143-159
26	WDMeV/N281/1071nt | WD08.18 | MT247856	14-635 | Q80	35425 | 0.44%	1071 nt long consensus sequence (partial PP)	+	1	<1	>1071	1071 | 356	WDMeV/4874nt/WD08.18: 99.2 % in aa (MEGA analysis)	RNA helicase: 277-357 aa of PP

Supplementary material 2

Metagenomic characterisation of avian parvoviruses and picornaviruses from Australian wild ducks

Running title:

Avian parvoviruses and picornaviruses in Australian wild ducks

Supplementary material 2:

Phylogenetic analysis of avian parvoviruses and picornaviruses in Australian wild ducks

Authors

Jessy Vibin*[1,2], Anthony Chamings[1,2], Marcel Klaassen[3], Tarka Raj Bhatta[1,2], Soren Alexandersen*[1,2,4]

1 Geelong Centre for Emerging Infectious Diseases, Geelong, VIC 3220, Australia;

2 Deakin University, School of Medicine, Geelong, VIC 3220, Australia;

3 Deakin University, Centre for Integrative Ecology, Waurn Ponds, VIC 3216, Australia;

4 Barwon Health, Geelong, VIC 3220, Australia;

*Corresponding Authors: jessyatall@gmail.com, soren.alexandersen@deakin.edu.au

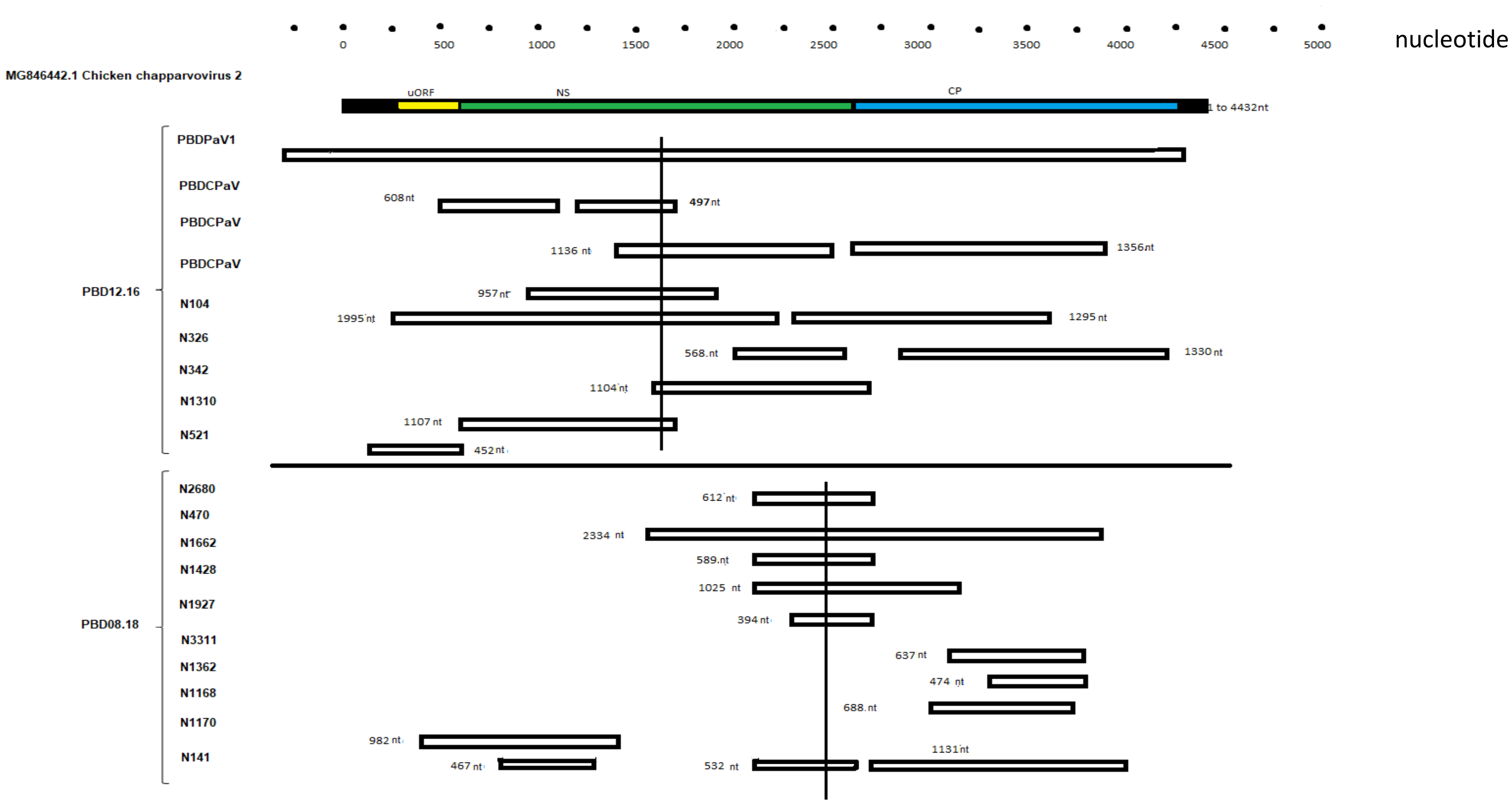

Figure S1: Chaphamaparvovirus (CPaV) sequences from Pacific black duck (PBD) samples
All the chaphamaparvoviruses sequences generated from the Pacific black duck samples are approximately aligned to the full-length MG846442 Chicken chaphamaparvovirus 2 virus sequence for comprehending the position of the generated partial genome consensus sequences and to determine the least number of chaphamaparvoviruses identified from each bird species. This figure provides the name of the virus sequence given, the general position of the sequence in the full-length genome of the virus and also shows the protein coded by the virus sequence. The vertical line along the PBDCPaV sequences enables to determine the minimum number of CPaV isolated and characterised from the duck species as these sequences are not identical to each other both in the nucleotide and amino acid level, as described. There are at least 7 chaphamaparvovirus present in PBD12.16 sample and 6 chaphamaparvovirus present in PBD08.18 sample. N represents the node/contig from which the sequence has been generated. uORF may or may not be present depending on the sequence

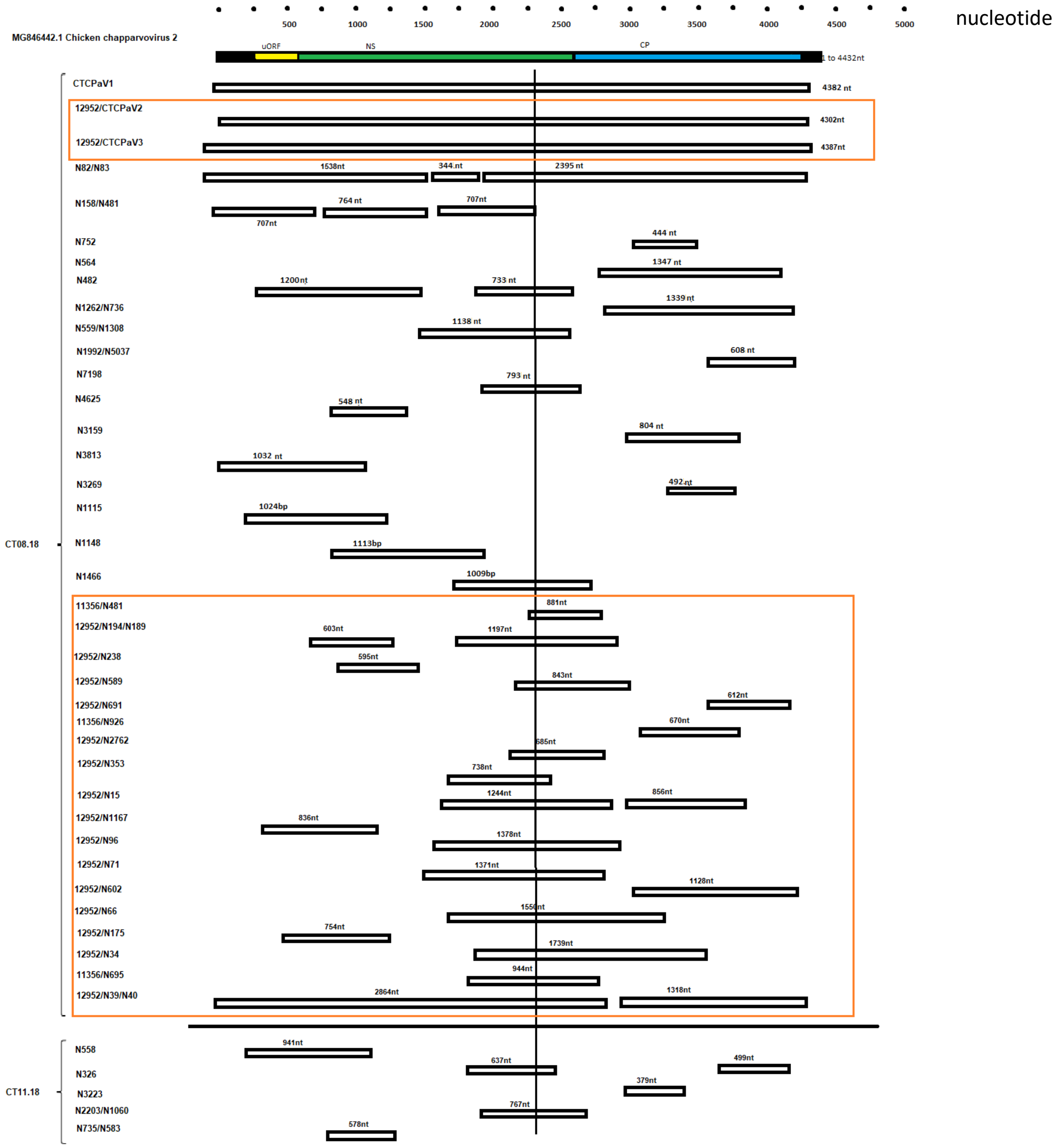

Figure S2: Chaphamaparvovirus (CPaV) sequences from Chestnut teal (CT) samples
All the chaphamaparvovirus sequences generated from the Chestnut teal samples are approximately aligned to the full-length MG846442 Chicken chaphamaparvovirus 2 virus sequence for comprehending the position of the generated partial genome consensus sequences and to determine the least number of chaphamaparvoviruses identified from each bird species. This figure provides the name of the virus sequence given, the general position of the sequence in the full-length genome of the virus and also shows the protein coded by the virus sequence. The vertical line along the CTCPaV sequences enables to determine the minimum number of CPaV isolated and characterised from the duck species as these sequences are not identical to each other both in the nucleotide and amino acid level, as described. There are at least 21 chaphamaparvovirus present in CT08.18 sample and 2 chaphamaparvovirus present in CT11.18 sample. N represents the node/contig from which the sequence has been generated. The viruses isolated from individual CT08.18 samples (11356 and 12952) are shown in the orange box. uORF may or may not be present depending on the sequence

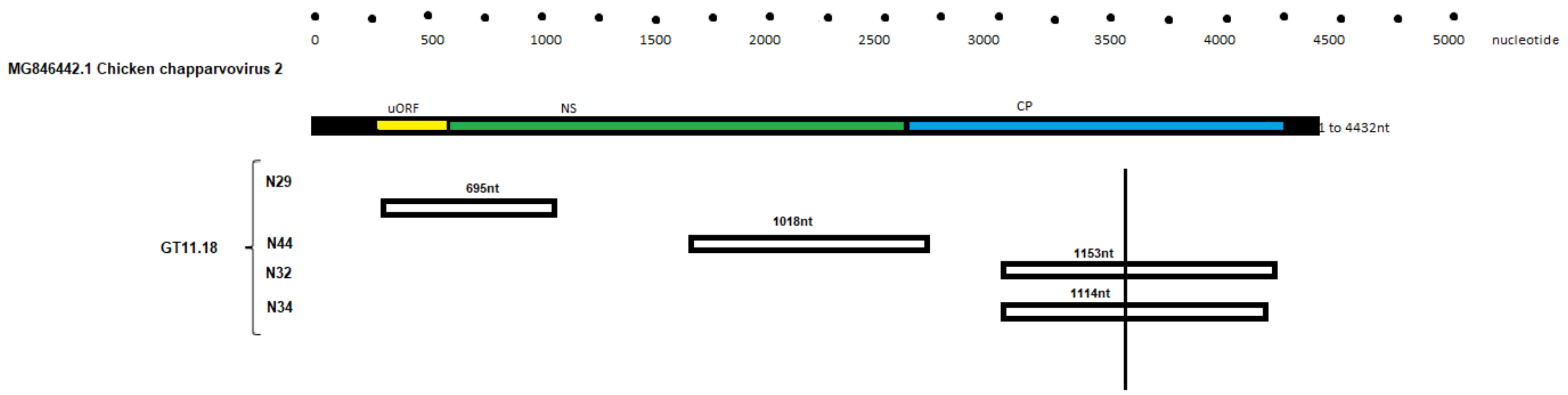

Figure S3: Chaphamaparvovirus (CPaV) sequences from Grey teal (CT) samples

All the chaphamaparvovirus sequences generated from the Grey teal samples are approximately aligned to the full-length MG846442 Chicken chaphamaparvovirus 2 virus sequence for comprehending the position of the generated partial genome consensus sequences and to determine the least number of chaphamaparvoviruses identified from each bird species. This figure provides the name of the virus sequence given, the general position of the sequence in the full-length genome of the virus and also shows the protein coded by the virus sequence. The vertical line along the GTCPaV sequences enables to determine the minimum number of CPaV isolated and characterised from the duck species as these sequences are not identical to each other both in the nucleotide and amino acid level, as described. There are 2 chaphamaparvovirus present in GT11.18 sample and 2 chaphamaparvovirus present in GT11.18 sample. N represents the node/contig from which the sequence has been generated. uORF may or may not be present depending on the sequence

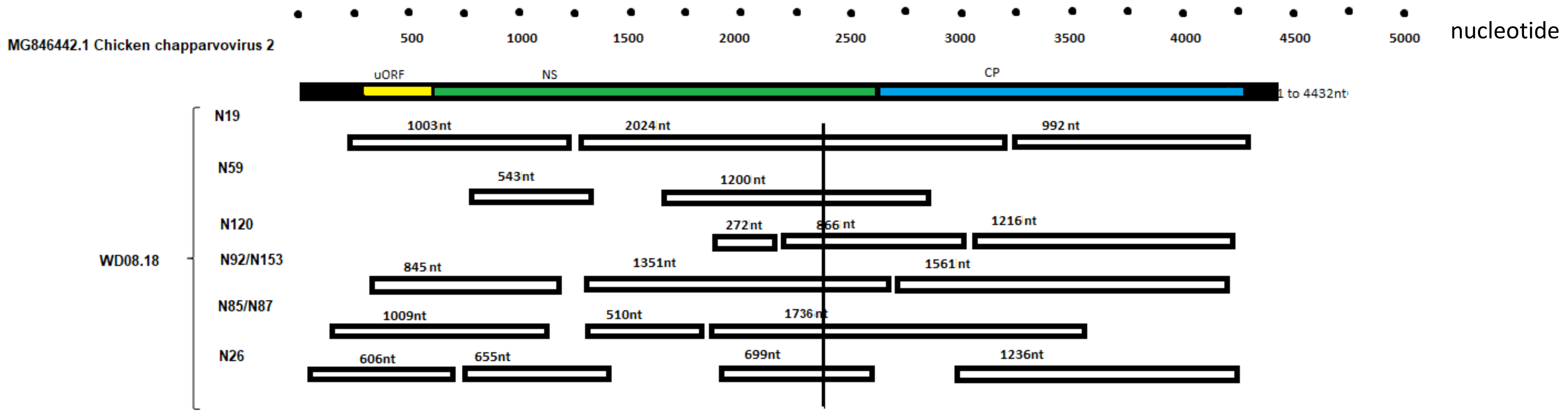

Figure S4: Chaphamaparvovirus (CPaV) sequences from Wood duck (WD) samples

All the chaphamaparvovirus sequences generated from the Wood duck samples are approximately aligned to the full-length MG846442 Chicken chaphamaparvovirus 2 virus sequence for comprehending the position of the generated partial genome consensus sequences and to determine the least number of chaphamaparvoviruses identified from each bird species. This figure provides the name of the virus sequence given, the general position of the sequence in the full-length genome of the virus and also shows the protein coded by the virus sequence. The vertical line along the WDCPaV sequences enables to determine the minimum number of CPaV isolated and characterised from the duck species as these sequences are not identical to each other both in the nucleotide and amino acid level, as described. There are 6 chaphamaparvovirus present in WD08.18 sample. N represents the node/contig from which the sequence has been generated. uORF may or may not be present depending on the sequence

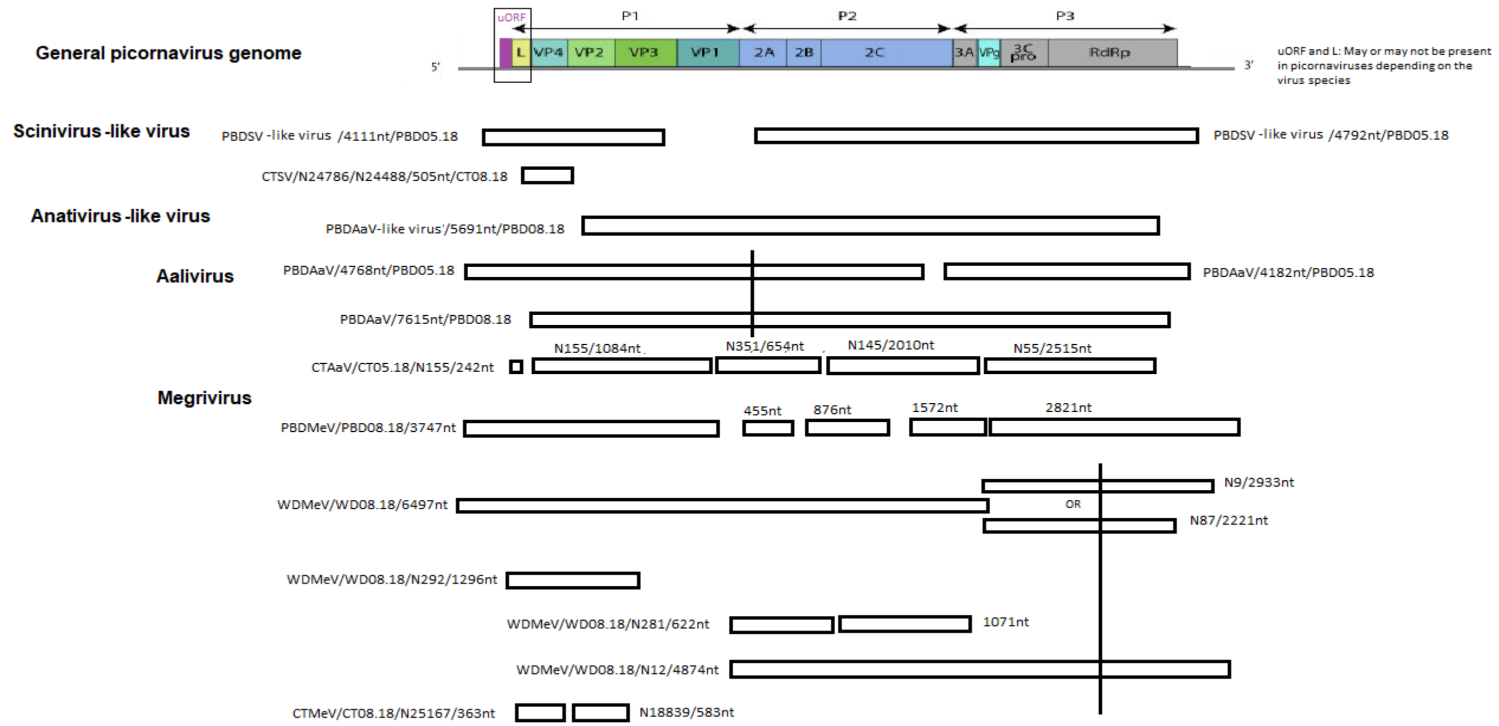

Figure S5: Picornavirus (PiV) sequences from the duck samples

All the picornavirus consensus sequences generated from the duck samples are approximately aligned to the general picornavirus genome organisation for comprehending the position of the generated partial genome consensus sequences. This figure provides the name of the virus sequence given, the genus they belong and the general position of the sequence in the full-length genome of the virus. The figure also enables to determine the minimum number of picornaviruses present in the bird species using the vertical line. We identified and characterised at least 2 scinivirus-like viruses, 1 anativirus-like virus, 3 aaliviruses and at least 4 megriviruses from Pacific black ducks, Chestnut teals and Wood ducks. WDMeV/WD08.18 sequences N9/2933nt and N87/2221nt have overlapping sequences to WDMeV/6497nt/WD08.18 sequence (see results for more details).

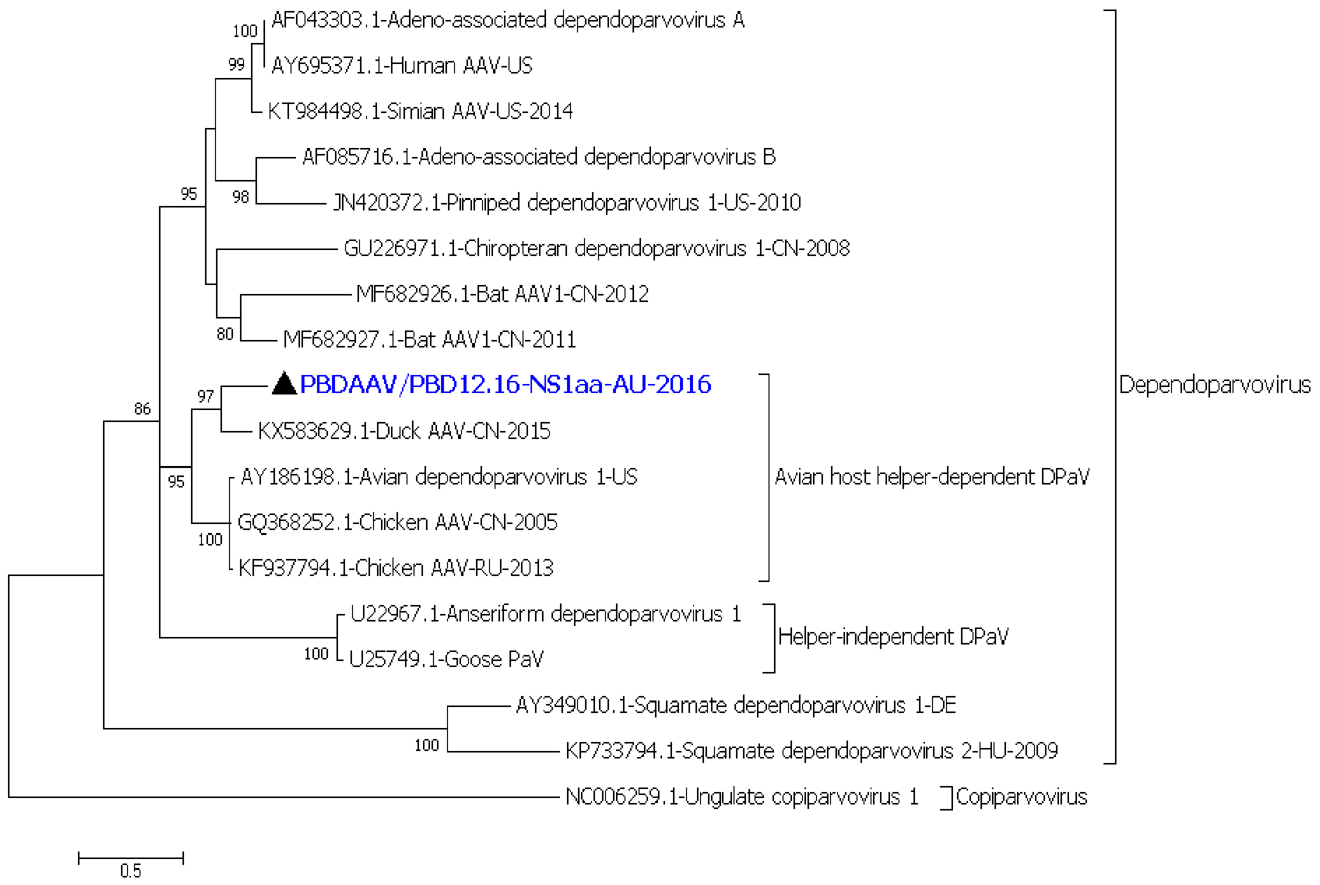

Figure S6: Phylogenetic analysis of NS1 amino acid sequence of Pacific black duck adeno-associated virus (PBDAAV/ PBD12.16)

The evolutionary history was inferred by using the Maximum Likelihood method based on the model LG+G+I[1]. The analysis involved 18 amino acid sequences. All positions containing gaps and missing data were eliminated. There were a total of 482 positions in the final dataset. The robustness of different nodes was assessed by bootstrap analysis using 1000 replicates for amino acid alignments. The numbers at the nodes represent bootstrap values and only bootstrap values at or above 60% are shown. Pacific black duck virus is shown with (▲). [DPaV: Dependoparvovirus]

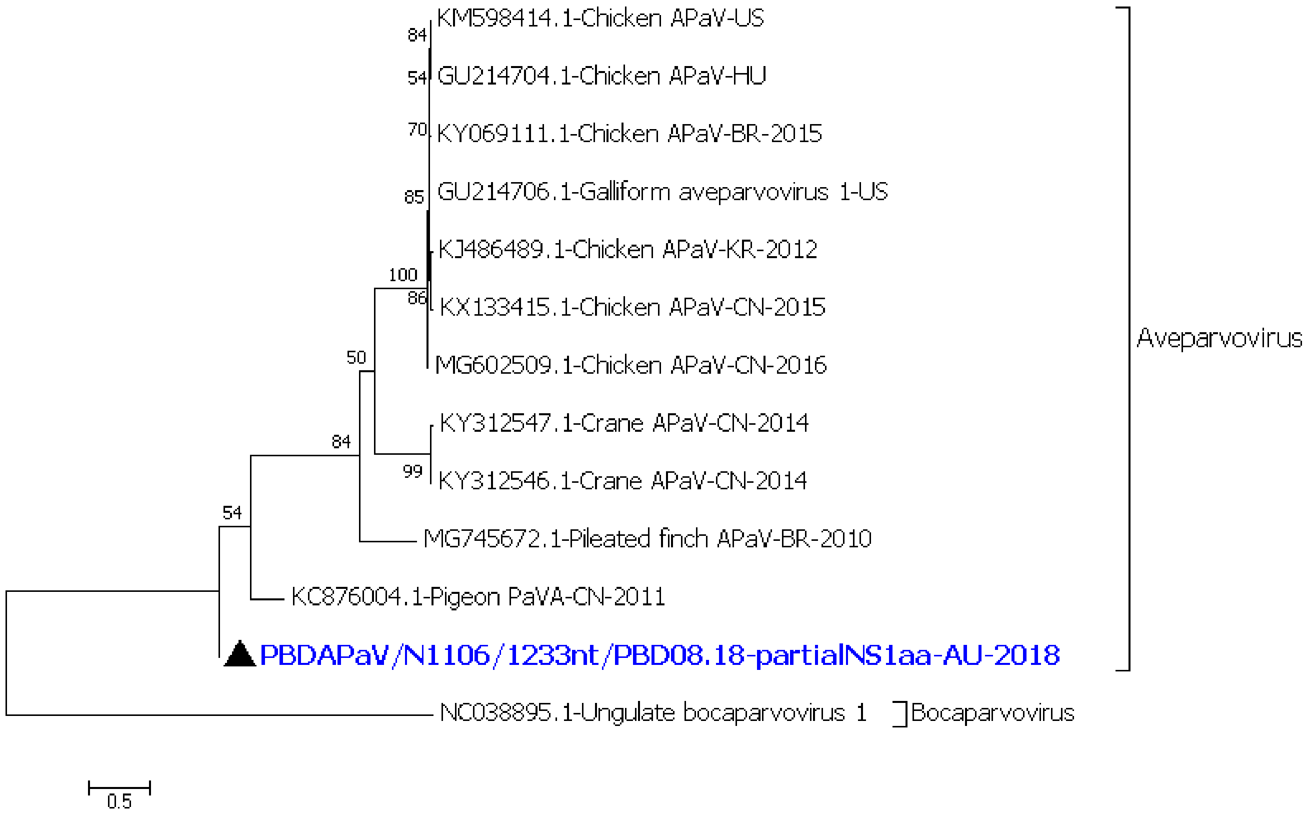

Figure S7: Phylogenetic analysis of the partial NS1 amino acid sequence of the Pacific black duck aveparvovirus (PBDAPaV/N1106/1233nt/PBD08.18) sequence

The evolutionary history was inferred by using the Maximum Likelihood method based on the model LG+G[1]. The analysis involved 13 amino acid sequences. All positions containing gaps and missing data were eliminated. There were a total of 363 positions in the final dataset. The robustness of different nodes was assessed by bootstrap analysis using 1000 replicates for amino acid alignments. The numbers at the nodes represent bootstrap values and only bootstrap values at or above 50% are shown. Pacific black duck virus is shown with (▲).

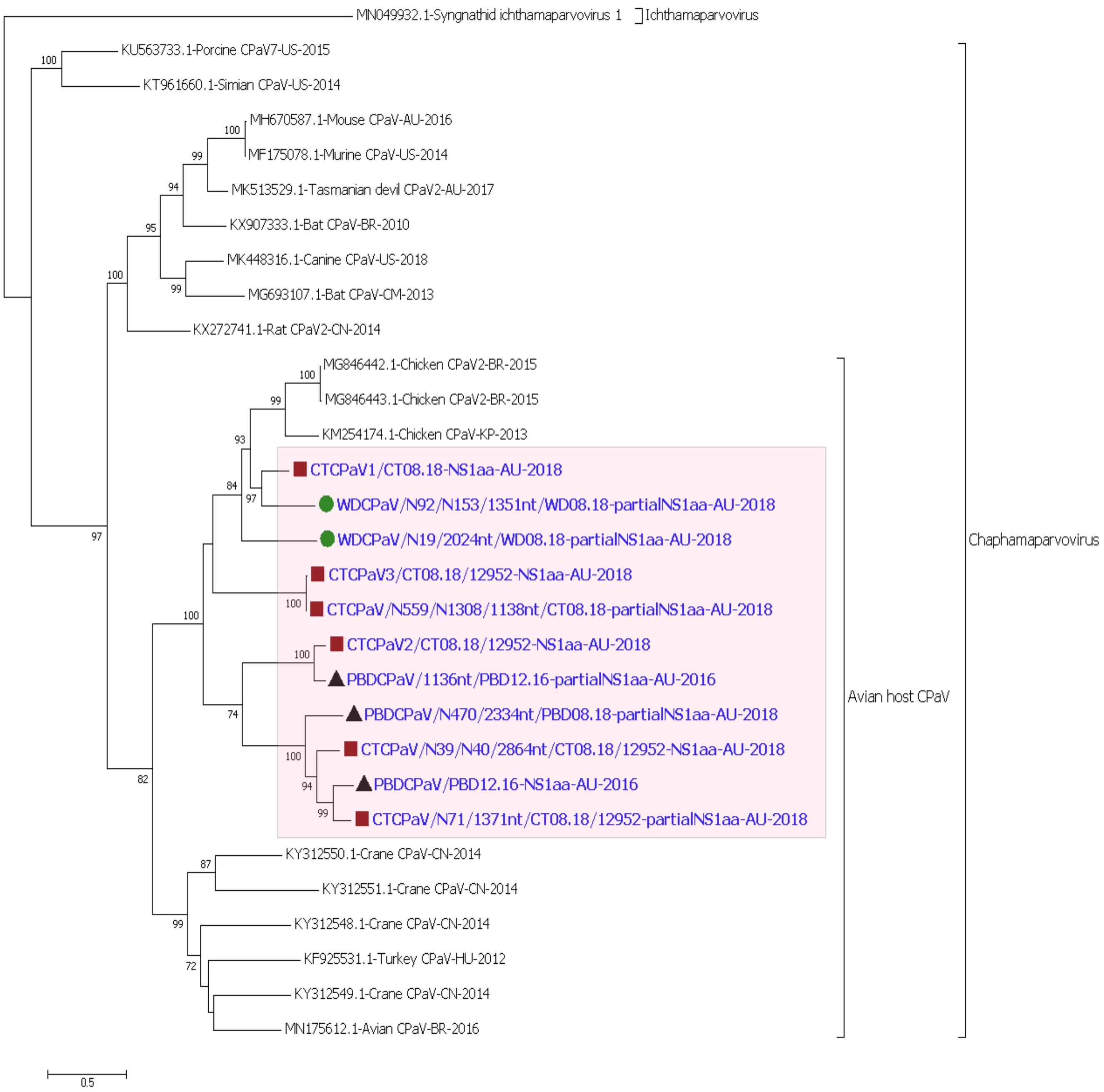

Figure S8: Phylogenetic analysis of helicase and partial NS1 region of the duck chaphamaparvovirus NS1 amino acid sequences showing duck lineages

The evolutionary history was inferred by using the Maximum Likelihood method based on the model JTT+G+I+F[2]. The analysis involved 30 amino acid sequences. All positions containing gaps and missing data were eliminated. There were a total of 270 positions in the final dataset. The robustness of different nodes was assessed by bootstrap analysis using 1000 replicates for amino acid alignments. The numbers at the nodes represent bootstrap values and only bootstrap values at or above 60% are shown. The highlighted section shows duck CPaV lineages/clusters. Pacific black duck viruses are shown with (▲), Chestnut teal viruses with (■) and Wood duck viruses with (●).

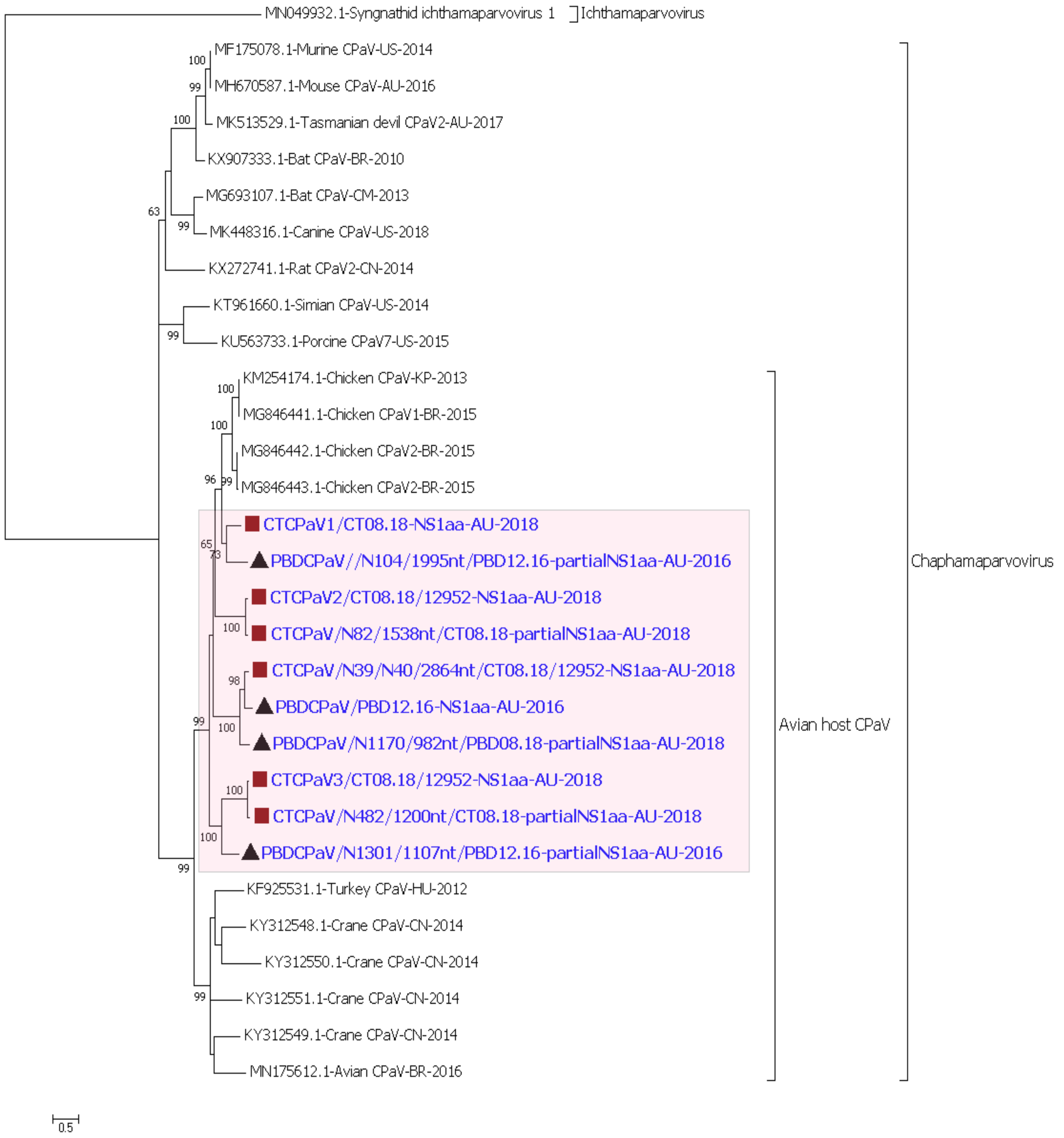

Figure S9: Phylogenetic analysis of the first part of the NS1 amino acid sequence of the duck chaphamaparvovirus sequences showing duck lineages

The evolutionary history was inferred by using the Maximum Likelihood method based on the model LG+G+I[1]. The analysis involved 30 amino acid sequences. All positions containing gaps and missing data were eliminated. There were a total of 232 positions in the final dataset. The robustness of different nodes was assessed by bootstrap analysis using 1000 replicates for amino acid alignments. The numbers at the nodes represent bootstrap values and only bootstrap values at or above 60% are shown. The highlighted section shows duck CPaV lineages/clusters. Pacific black duck viruses are shown with (▲) and Chestnut teal viruses with (■).

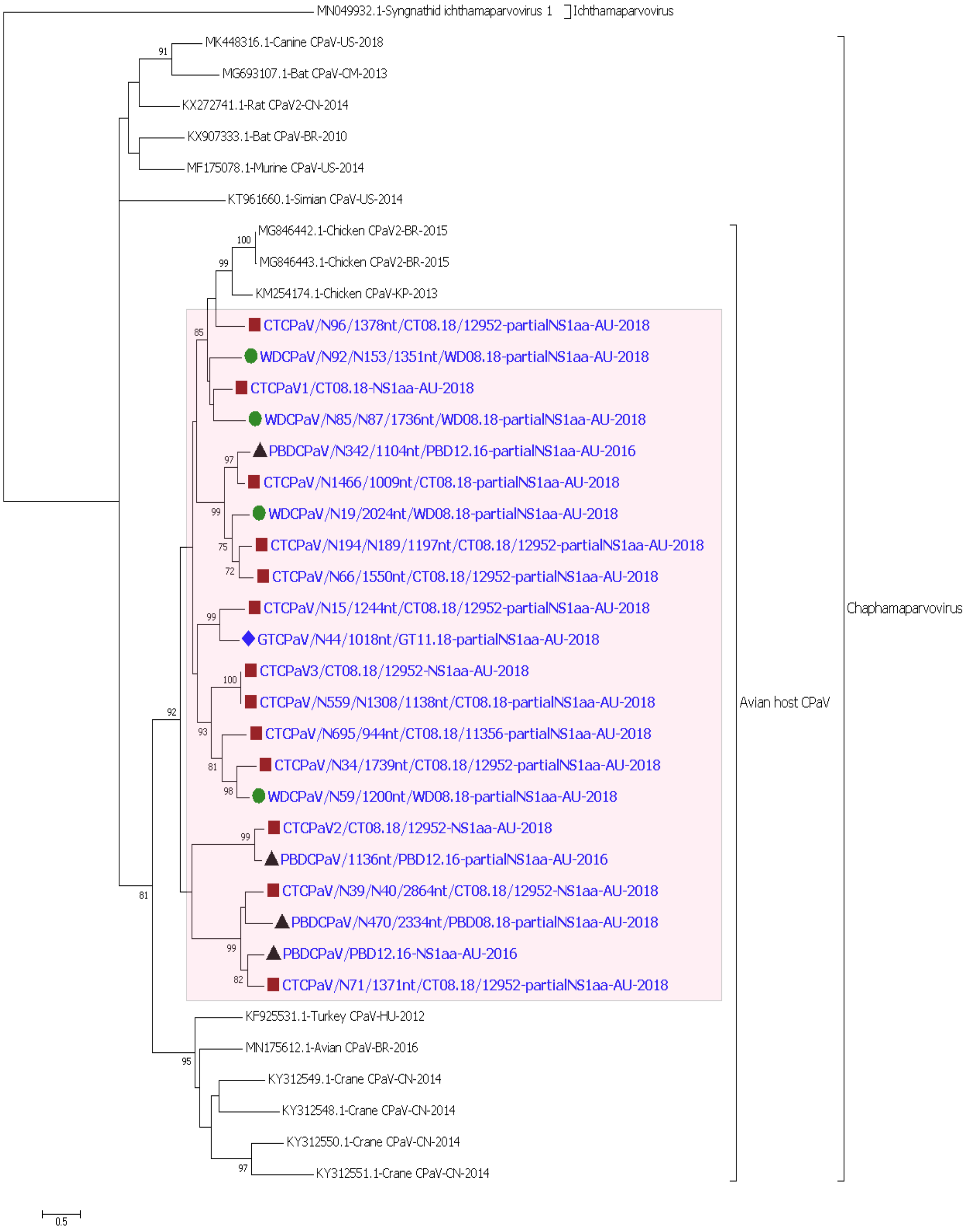

MN049932.1-Syngnathid ichthamaparvovirus 1
Ichthamaparvovirus
MK448316.1-Canine CPaV-US-2018
MG693107.1-Bat CPaV-CM-2013
KX272741.1-Rat CPaV2-CN-2014
KX907333.1-Bat CPaV-BR-2010
MF175078.1-Murine CPaV-US-2014
KT961660.1-Simian CPaV-US-2014
MG846442.1-Chicken CPaV2-BR-2015
MG846443.1-Chicken CPaV2-BR-2015
KM254174.1-Chicken CPaV-KP-2013
CTCPaV/N96/1378nt/CT08.18/12952-partialNS1aa-AU-2018
WDCPaV/N92/N153/1351nt/WD08.18-partialNS1aa-AU-2018
CTCPaV1/CT08.18-NS1aa-AU-2018
WDCPaV/N85/N87/1736nt/WD08.18-partialNS1aa-AU-2018
PBDCPaV/N342/1104nt/PBD12.16-partialNS1aa-AU-2016
CTCPaV/N1466/1009nt/CT08.18-partialNS1aa-AU-2018
WDCPaV/N19/2024nt/WD08.18-partialNS1aa-AU-2018
CTCPaV/N194/N189/1197nt/CT08.18/12952-partialNS1aa-AU-2018
CTCPaV/N66/1550nt/CT08.18/12952-partialNS1aa-AU-2018
CTCPaV/N15/1244nt/CT08.18/12952-partialNS1aa-AU-2018
GTCPaV/N44/1018nt/GT11.18-partialNS1aa-AU-2018
CTCPaV3/CT08.18/12952-NS1aa-AU-2018
CTCPaV/N559/N1308/1138nt/CT08.18-partialNS1aa-AU-2018
CTCPaV/N695/944nt/CT08.18/11356-partialNS1aa-AU-2018
CTCPaV/N34/1739nt/CT08.18/12952-partialNS1aa-AU-2018
WDCPaV/N59/1200nt/WD08.18-partialNS1aa-AU-2018
CTCPaV2/CT08.18/12952-NS1aa-AU-2018
PBDCPaV/1136nt/PBD12.16-partialNS1aa-AU-2016
CTCPaV/N39/N40/2864nt/CT08.18/12952-NS1aa-AU-2018
PBDCPaV/N470/2334nt/PBD08.18-partialNS1aa-AU-2018
PBDCPaV/PBD12.16-NS1aa-AU-2016
CTCPaV/N71/1371nt/CT08.18/12952-partialNS1aa-AU-2018
KF925531.1-Turkey CPaV-HU-2012
MN175612.1-Avian CPaV-BR-2016
KY312549.1-Crane CPaV-CN-2014
KY312548.1-Crane CPaV-CN-2014
KY312550.1-Crane CPaV-CN-2014
KY312551.1-Crane CPaV-CN-2014
Avian host CPaV
Chaphamaparvovirus
0.5

Figure S10: Phylogenetic analysis of the last part of the NS1 amino acid sequence of the Chaphamaparvovirus sequences showing duck lineages

The duck chaphamaparvoviruses formed duck clusters/lineages separate to chaphamaparvoviruses from other avian hosts. The evolutionary history was inferred by using the Maximum Likelihood method based on the model JTT+G+F[2]. The analysis involved 38 amino acid sequences. All positions containing gaps and missing data were eliminated. There were a total of 168 amino acid positions in the final dataset. The robustness of different nodes was assessed by bootstrap analysis using 1000 replicates for amino acid alignments. The numbers at the nodes represent bootstrap values and only bootstrap values at or above 60% are shown. The highlighted section shows duck CPaV lineages/clusters. Pacific black duck viruses are shown with (▲), Chestnut teal viruses with (■), Wood duck viruses with (●) and Grey teal viruses with (◆).

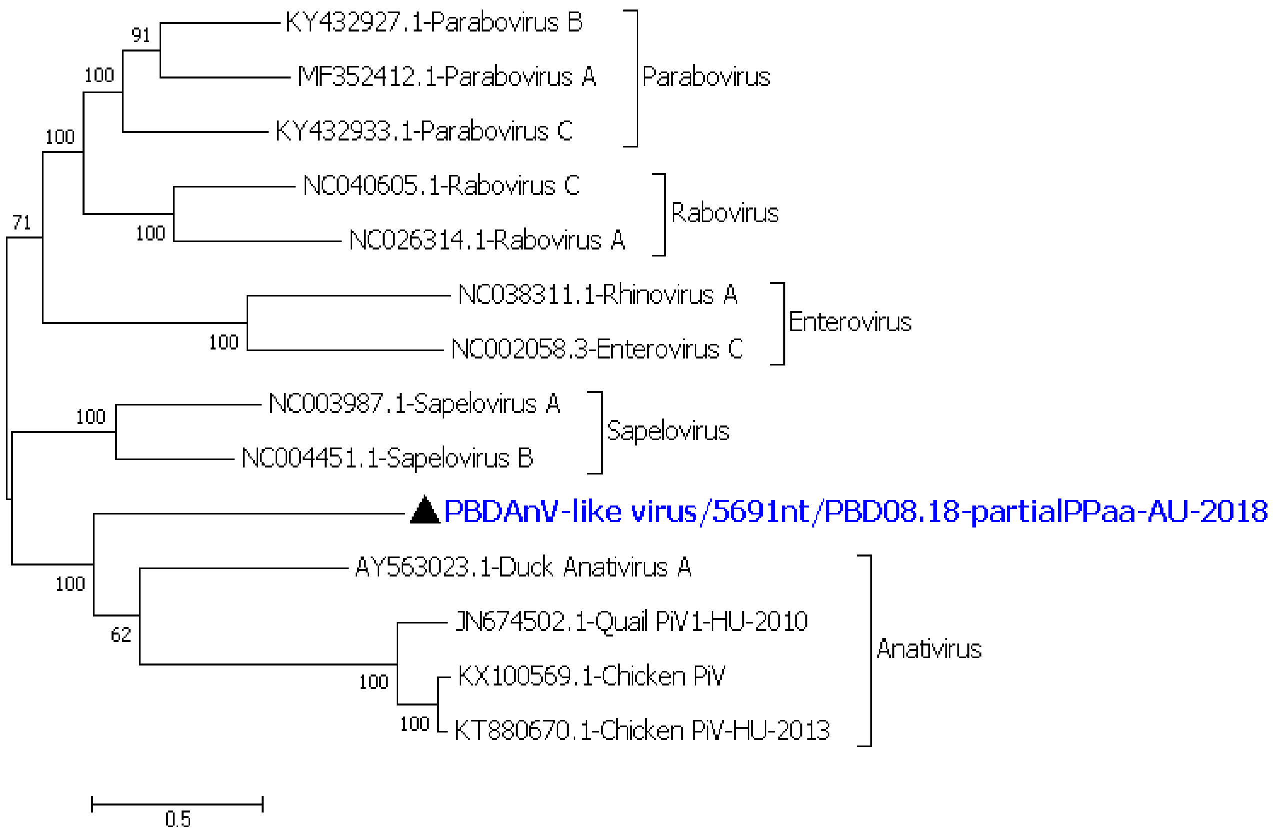

Figure S11: Phylogenetic analysis of the amino acid sequence of the polyprotein of Pacific black duck anativirus-like virus (PBDAnV-like virus/5691nt/PBD08.18) sequence

The evolutionary history was inferred by using the Maximum Likelihood method based on the model LG+G+I+F[1]. The analysis involved 14 amino acid sequences. All positions containing gaps and missing data were eliminated. There were a total of 1539 positions in the final dataset. The robustness of different nodes was assessed by bootstrap analysis using 1000 replicates for amino acid alignments. The numbers at the nodes represent bootstrap values and only bootstrap values at or above 60% are shown. Pacific black duck virus is shown in (▲).

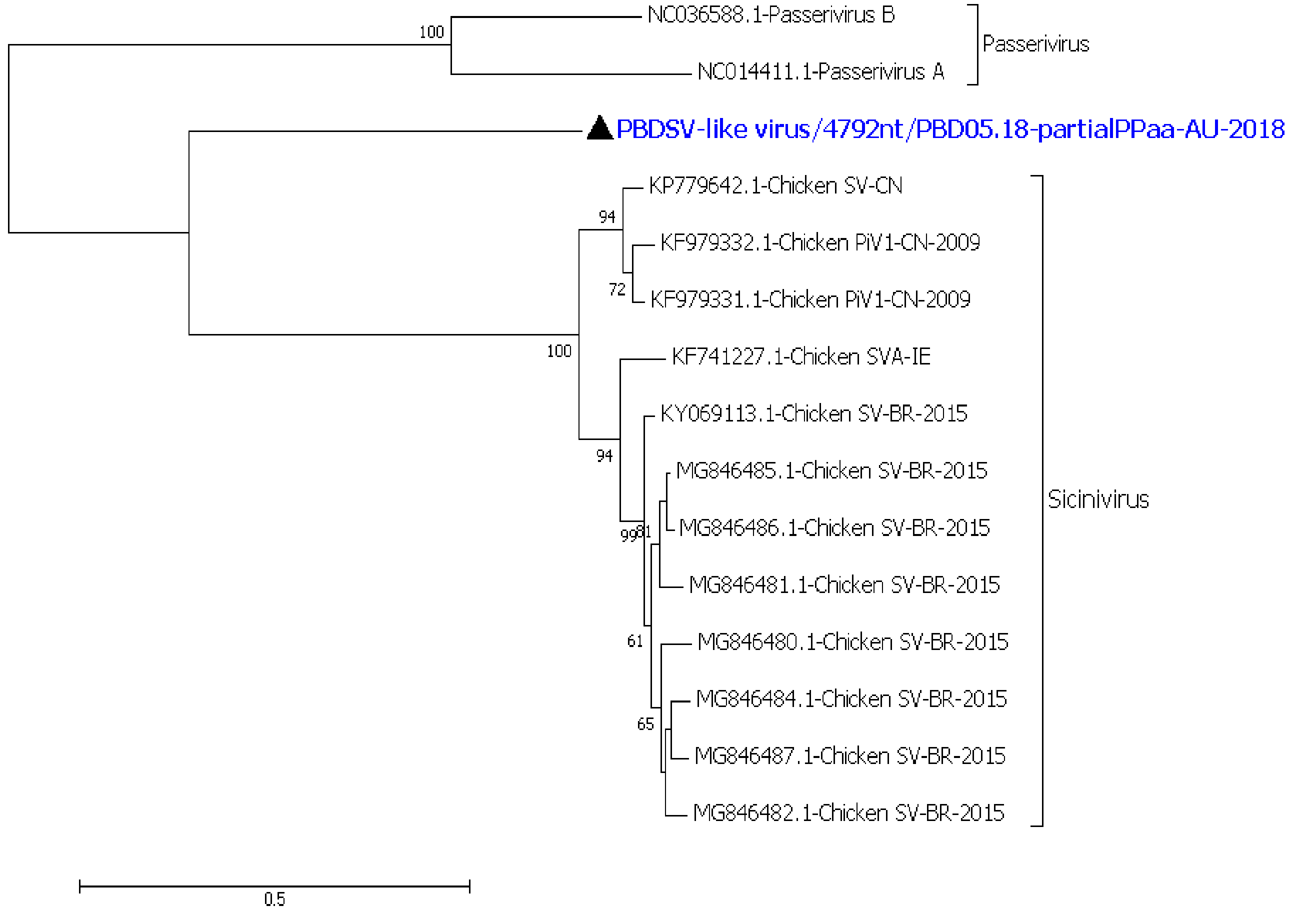

Figure S12: Phylogenetic analysis of the amino acid sequence of the Pacific black duck sicinivirus-like virus (PBDSV-like virus/4792nt/PBD05.18) sequence encoding the RdRp, Peptidase C3 and Helicase region

The evolutionary history was inferred by using the Maximum Likelihood method based on the model LG+G[1]. The analysis involved 15 amino acid sequences. All positions containing gaps and missing data were eliminated. There were a total of 1356 positions in the final dataset. The robustness of different nodes was assessed by bootstrap analysis using 1000 replicates for amino acid alignments. The numbers at the nodes represent bootstrap values and only bootstrap values at or above 60% are shown. Pacific black duck virus is shown in (▲).

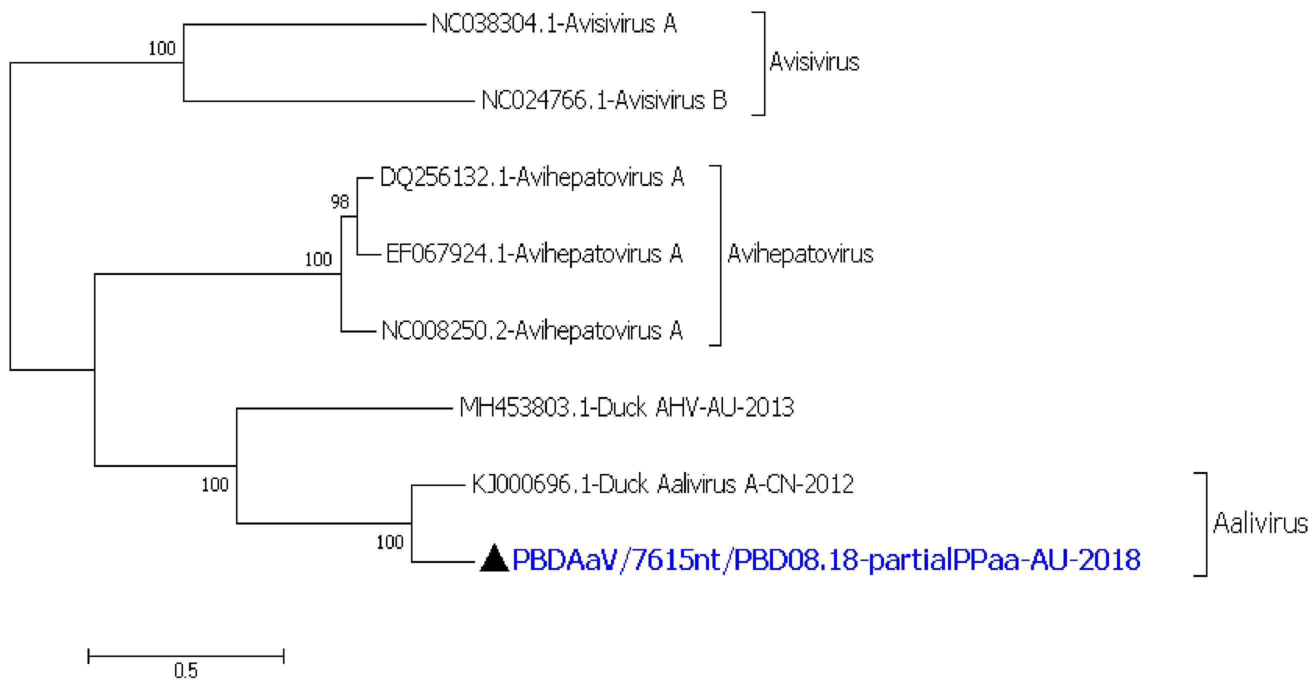

Figure S13: Phylogenetic analysis of the amino acid sequence of the polyprotein of Pacific black duck aalivirus (PBDAaV/7615nt/PBD08.18) sequence

The evolutionary history was inferred by using the Maximum Likelihood method based on the model LG+G[1]. The analysis involved 8 amino acid sequences. All positions containing gaps and missing data were eliminated. There were a total of 1928 positions in the final dataset. Only Duck AaV-CN-2010 (GenBank accession number KJ000696) along with PBDAaV and CTAaV belongs to the *Aalivirus* genus. The robustness of different nodes was assessed by bootstrap analysis using 1000 replicates for amino acid alignments. MH453803.1 Duck Avihepatovirus-like virus is an unassigned virus. The numbers at the nodes represent bootstrap values and only bootstrap values at or above 60% are shown. Pacific black duck virus is shown in (▲).

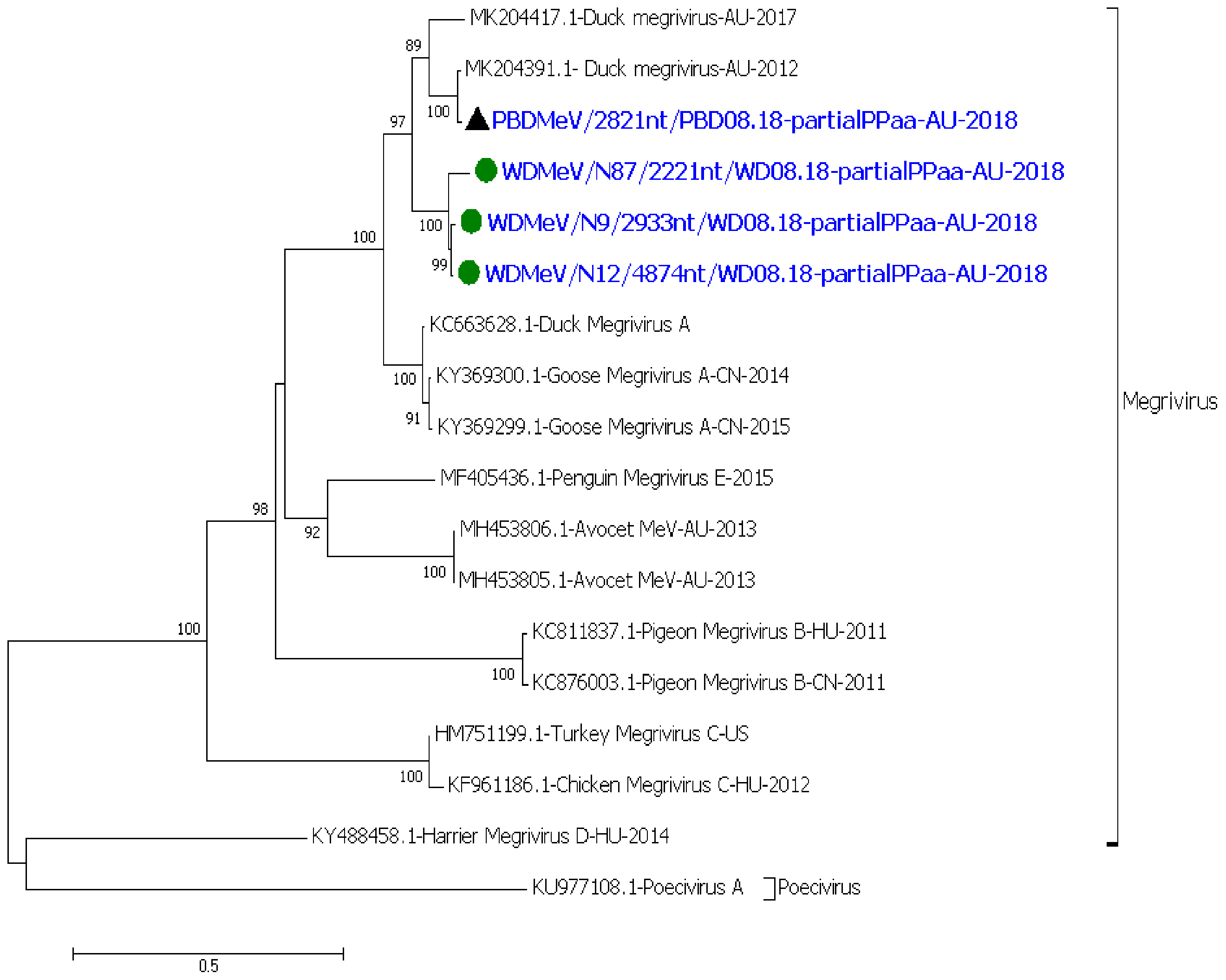

Figure S14: Phylogenetic analysis of the amino acid sequence of the RdRp and Peptidase C3 of duck megrivirus (MeV) sequences

The evolutionary history was inferred by using the Maximum Likelihood method based on the model LG+G+I[1]. The analysis involved 18 amino acid sequences. All positions containing gaps and missing data were eliminated. There were a total of 526 positions in the final dataset. The robustness of different nodes was assessed by bootstrap analysis using 1000 replicates for amino acid alignments. The numbers at the nodes represent bootstrap values and only bootstrap values at or above 60% are shown. Pacific black duck virus is shown in (▲) and Wood duck viruses in (●).

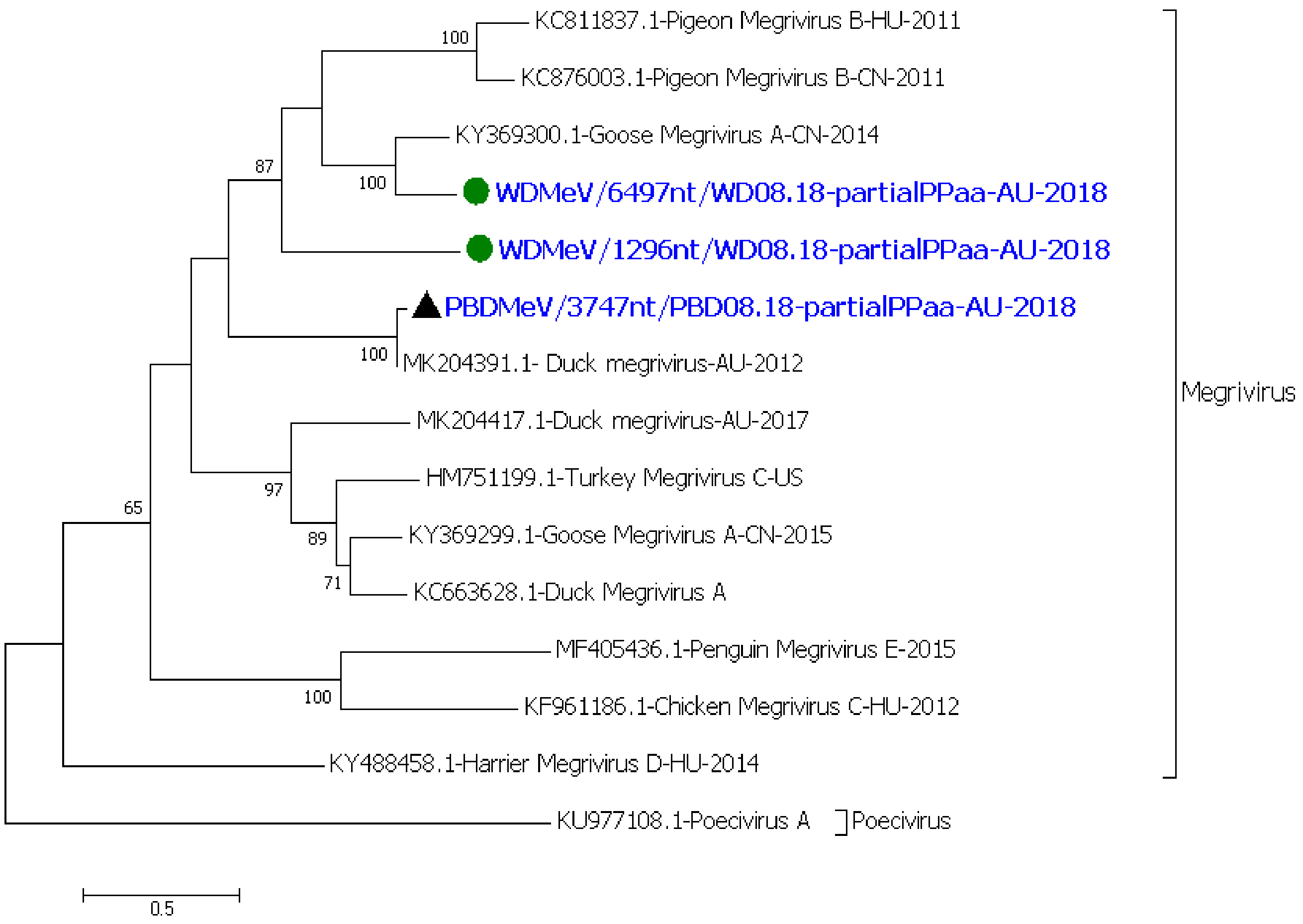

Figure S15: Phylogenetic analysis of the amino acid sequence of the polyprotein encoding the capsid region of duck megrivirus sequences

The evolutionary history was inferred by using the Maximum Likelihood method based on the model LG+G+F[1]. The analysis involved 15 amino acid sequences. All positions containing gaps and missing data were eliminated. There were a total of 383 positions in the final dataset. The robustness of different nodes was assessed by bootstrap analysis using 1000 replicates for amino acid alignments. The numbers at the nodes represent bootstrap values and only bootstrap values at or above 60% are shown. Pacific black duck virus is shown in (▲) and Wood duck viruses in (●).

1. Le, S. Q. & Gascuel, O. An Improved General Amino Acid Replacement Matrix. *Mol. Biol. Evol.* **25**, 1307–1320 (2008).
2. Jones, D. T., Taylor, W. R. & Thornton, J. M. The rapid generation of mutation data matrices from protein sequences. *Comput. Appl. Biosci.* **8**, 275–82 (1992).
